# An Isolable 2,5‐Disila‐3,4‐Diphosphapyrrole and a Conjugated Si=P−Si=P−Si=N Chain Through Degradation of White Phosphorus with a *N,N*‐Bis(Silylenyl)Aniline

**DOI:** 10.1002/anie.202209250

**Published:** 2022-08-08

**Authors:** Yun Xiong, Shicheng Dong, Shenglai Yao, Chenshu Dai, Jun Zhu, Sebastian Kemper, Matthias Driess

**Affiliations:** ^1^ Department of Chemistry: Metalorganic and Inorganic Materials Technische Universität Berlin Strasse des 17. Juni 135, Sekr. C2 10623 Berlin Germany; ^2^ State Key Laboratory of Physical Chemistry of Solid Surface and Collaborative Innovation Center of Chemistry for Energy Materials (iChEM) and College of Chemistry and Chemical Engineering Xiamen University Xiamen 361005 P. R. China

**Keywords:** Metal-Free Bond Activation, Non-Aromatic Heterocycles, Phosphorus, Silicon

## Abstract

White phosphorus (P_4_) undergoes degradation to P_2_ moieties if exposed to the new *N,N*‐bis(silylenyl)aniline PhN**Si**_2_ **1** (**Si**=Si[N(*t*Bu)]_2_CPh), furnishing the first isolable 2,5‐disila‐3,4‐diphosphapyrrole **2** and the two novel functionalized Si=P doubly bonded compounds **3** and **4**. The pathways for the transformation of the non‐aromatic 2,5‐disila‐3,4‐diphosphapyrrole PhN**Si**_2_P_2_ **2** into **3** and **4** could be uncovered. It became evident that **2** reacts readily with both reactants P_4_ and **1** to afford either the polycyclic Si=P‐containing product [PhN**Si**_2_P_2_]_2_P_2_ **3** or the unprecedented conjugated **Si**=P−**Si**=P−**Si**=NPh chain‐containing compound **4**, depending on the employed molar ratio of **1** and P_4_ as well as the reaction conditions. Compounds **3** and **4** can be converted into each other by reactions with **1** and P_4_, respectively. All new compounds **1**–**4** were unequivocally characterized including by single‐crystal X‐ray diffraction analysis. In addition, the electronic structures of **2**–**4** were established by Density Functional Theory (DFT) calculations.

## Graphical abstract

A novel geminal *N*,*N*‐bis(silylenyl)aniline **1** degrades white phosphorus (P_4_) furnishing the first isolable 2,5‐disila‐3,4‐ diphosphapyrrole **2**. The latter further reacts readily with P_4_ and **1** to afford the polycyclic Si=P‐containing species **3** or the unprecedented conjugated Si=P−Si=P−Si=NPh chain‐containing compound **4** depending on the molar ratio of the reactants. Moreover, the products **3** and **4** can be converted into each other when treated with **1** and P_4_, respectively.

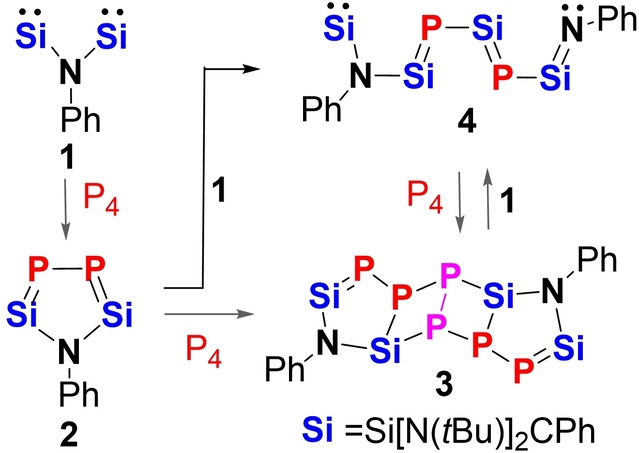

## Introduction

Multiply bonded heavy element main‐group chemistry has experienced a rapid development since the landmark discovery from West and co‐workers of the first isolable disilene with a discrete Si=Si bond and from Yoshifuji and co‐workers of the synthesis of an isolable diphosphene featuring a discrete P=P bond in 1981.^[1]^ These systems broke the “double‐bond rule” for elements of the periodic table with a principal quantum number greater than 2.^[2]^ Due to their different bonding situation in comparison to E−E multiple bonds between second row p‐block elements and hence their unique electronic properties and reactivities, multiply bonded chemical species involving heavy main group elements,^[2]^ especially those between Group 14 and 15 elements,^[2g]^ have established a new area in main‐group chemistry. Even mixed Group 14 and 15 element E=E′‐containing compounds could be realized. Among them, doubly bonded Si=P compounds, called phosphasilenes, remained elusive for many years. The first example of a Si=P containing species was characterized with ^29^Si and ^31^P NMR spectroscopy in 1984 by Bickelhaupt et al.;^[3a]^ almost a decade later, the first structurally characterized phosphasilene was reported by Niecke and co‐workers in 1993.^[3b]^ The synthetic approach initially used to isolate phosphasilenes was the reaction of aryl‐PHLi with R_2_SiCl_2_ with the elimination of LiCl and aryl‐PH_2_.^[3]^ By employing a similar strategy, an effective pathway for synthesizing *P*‐silyl‐substituted phosphasilenes, via the elimination of LiF or Me_3_SiCl, was developed in our group.^[4]^ A very different approach to phosphasilene derivatives was developed in 2011 via the direct activation of white phosphorus (P_4_) by three‐coordinate *N*‐heterocyclic silylenes (NHSi)s. Some remarkable examples include the activation of P_4_ by the silylene amide **Si**N(SiMe_3_)_2_ (**Si**=Si[N(*t*Bu)]_2_CPh)^[5]^ and the silylene‐phosphine CB**SiP** ^[6]^ (CB=*ortho*‐C,C′‐C_2_B_10_H_10_; **P**=P[N(*t*Bu)CH_2_]_2_), yielding phosphasilenes featuring a P_4_‐ and P_5_‐chain, respectively. Quite recently, a phosphasilene with a P_3_ chain was obtained via the further activation of a P_4_ activation product L′SiP_4_ ^[7]^ (L′=CH[C(Me)N(Dipp)][C(CH_2_)N(Dipp)], Dipp=2,6‐*i*Pr_2_C_6_H_3_) by a three‐coordinate silylene **Si**−N(Me)(2‐pyridine).^[8a]^ In addition, two phosphasilenes with P_2_ chains were reported via P_4_ activation by two metallasilylenes **Si**−M(Cl)L (M=Al, Ga; L=CH(CMeN(Dipp)_2_).^[8b]^ In these examples, two or three mono‐silylene moieties co‐activate one P_4_ molecule, furnishing Si=P containing species with incompletely activated P_*n*_ chains (*n*=2–5).

**Scheme 1:**
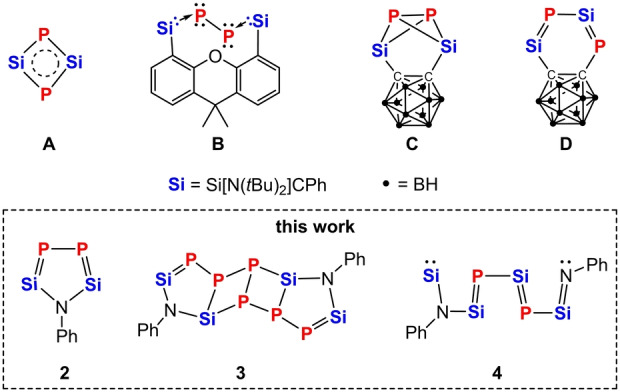
Examples of the white phosphorus activation products **A**–**D** and the new compounds **2**, **3**, and **4** described in this work.

**Scheme 2:**
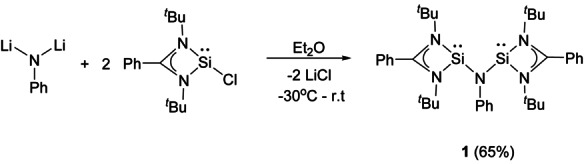
Synthesis of the *N*,*N*‐bis(silylenyl)aniline **1**.

Remarkably, the aromatic phosphasilene **A** ^[9a]^ (Scheme 1) was obtained from P_4_ activation by the disilylene **Si**−**Si** in 2011.^[9b]^ In line with this result, utilizing bis(silylenes) with different backbones (spacers), we recently achieved the synthesis of the bis(silylene)‐supported P_2_ species **B** ^[10]^ and the Si_2_P_2_ butterfly‐like species **C** ^[6]^ via the activation of P_4_ with (Xant)**Si**_2_ (Xant=9,9‐dimethyl‐1,8‐diylxanthene)^[11]^ and CB**Si**_2_,^[12]^ respectively. Compound **B** represents a heavier homologue of (cAAC)_2_P_2_ ^[13a]^ and (NHC)_2_P_2_ ^[13b]^ (cAAC=cyclic alkyl(amino)carbene, NHC=*N*‐heterocyclic carbene) but features a distinctively different electronic structure. The butterfly‐like compound **C** is less stable and rearranges to the valence isomer **D** with a conjugated Si=P−Si=P moiety as final product. The latter results indicate that the outcome of P_4_ activation with bis(silylenes) is dependent on the distance of the Si^II^−Si^II^ moieties and the electronic nature of the spacer. We have now learnt that the bis(silylenyl)aniline **1** (Scheme 2) representing a new geminal bis(silylene), shows a strikingly different reaction mode towards P_4_. Herein we report on this new P_4_ activation pattern which allowed us to isolate the first non‐aromatic 2,5‐disila‐3,4‐diphosphapyrrole PhN**Si**_2_P_2_ **2**, the polycyclic Si=P‐containing compound [PhN**Si**_2_P_2_]_2_P_2_ **3**, and the first silylene‐bis(phosphasilene)‐silaimine **4**, featuring a conjugated :**Si**−N(Ph)−**Si**=P−**Si**=P−**Si**=NPh chain (Scheme 1).

## Results and Discussion

### Synthesis and Characterization of the Bis(silylenyl)aniline 1

The new bis(silylene) PhN**Si_2_ 1** is easily accessible in 65 % yields by salt‐metathesis reaction of dilithiated aniline^[14a]^ with **Si**Cl^[14b]^ in the molar ratio of 1 : 2 (Scheme 2).

**Figure 1:**
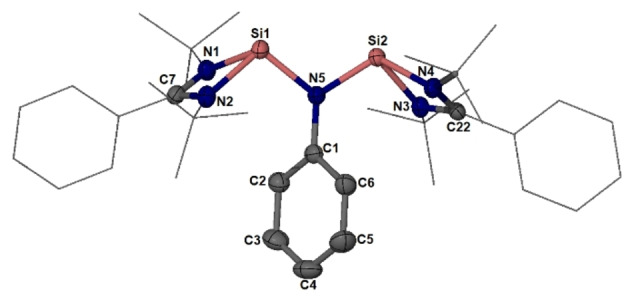
Molecular structure of **1**.^[20]^ Thermal ellipsoids are drawn at 50 % probability level. H atoms are omitted for clarity. Selected interatomic distances [Å] and angles [°]: Si1−Si2 2.895(1), Si2−N5 1.765(1), Si2−N3 1.875(1), Si2−N4 1.893(1), Si1−N5 1.781(1), Si1−N2 1.881(1), Si1−N1 1.908(1), N5−Si2−N3 102.9(1), N5−Si2−N4 106.2(1), N5−Si1−N2 101.6(1), N5−Si1−N1 105.0(1), C1−N5−Si2 124.5(1), C1−N5−Si1 126.0(1), Si2−N5−Si1 109.4(1).

The molecular structure of **1** has been unequivocally elucidated by NMR spectroscopy and single‐crystal X‐ray diffraction analysis (XRD). A singlet is observed at *δ*=−12.9 ppm in the ^29^Si{^1^H} NMR spectrum, akin to the chemical shift of the isoelectronic O**Si**_2_ (*δ*=−16.1 ppm).^[15]^ This is in contrast to the less shielded ^29^Si nucleus in the C‐substituted bis‐silylenes (Xant)**Si**_2_ (*δ*=17.9 ppm),^[11]^ and CB**Si**_2_ (*δ*=18.9 ppm).^[12]^ Compound **1** crystallized in the monoclinic space group *P*2_1_/*c* (Figure 1). The bridging N5 atom is trigonal‐planar coordinated with the sum of bond angles very close to 360° (∑=359.9°), implying delocalization of the lone pair of electrons at the nitrogen atom. Notably, the Si1−N5−Si2 angle of 109.4(1)° in **1** is significantly smaller than that observed in O**Si**_2_ (Si−O−Si angle: 159.9°).^[15]^ Accordingly, the intramolecular Si^II^⋅⋅⋅Si^II^ distance in **1** amounts to 2.895(1) Å, which is significantly shorter than those in O**Si**_2_ (3.243(1) Å),^[15]^ CB**Si**_2_ (3.2666(6) Å),^[12]^ and (Xant)**Si**_2_ (4.3155(9) Å).^[11]^

### Reaction of 1 with White Phosphorus

**Scheme 3:**
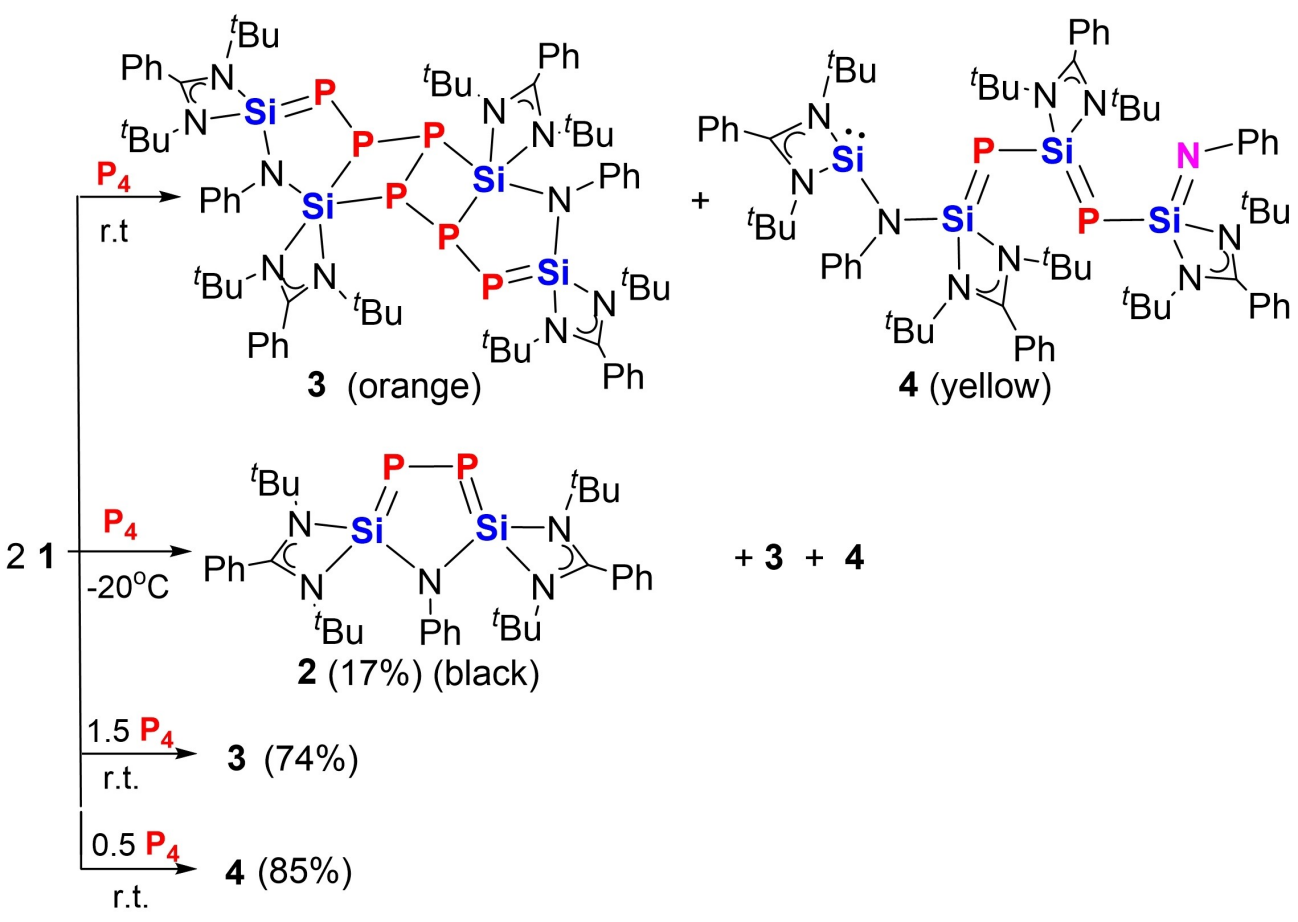
Synthesis of **2**, **3**, and **4** from **1** and P_4_.

Considering the formation and constitution of compounds **A**–**D**, bis(silylene) **1** and P_4_ were treated at first in a molar ratio of 2 : 1 at room temperature in Et_2_O or THF, affording, however, a mixture of the polycyclic compound [PhN**Si**_2_P_2_]_2_P_2_ **3** and compound **4** with a conjugated :**Si**−N(Ph)−**Si**=P−**Si**=P−**Si**=NPh chain (Scheme 3). Through fractional crystallization **3** and **4** could be separated. The successful identification of **3** and **4** by means of single‐crystal X‐ray diffraction analysis directed us to synthesize each compound individually according to their constituents. Accordingly, the reaction of **1** with P_4_ in a molar ratio of 2 : 1.5 at room temperature led to the quantitative formation of **3** as orange crystals in 74 % isolated yields (Scheme 3). On the other hand, the reaction of **1** with P_4_ in a molar ratio of 2 : 0.5 afforded **4** in high isolated yields (85 %) under similar reaction conditions (Scheme 3).

### Synthesis of 2 and Its Reactivity

**Scheme 4:**
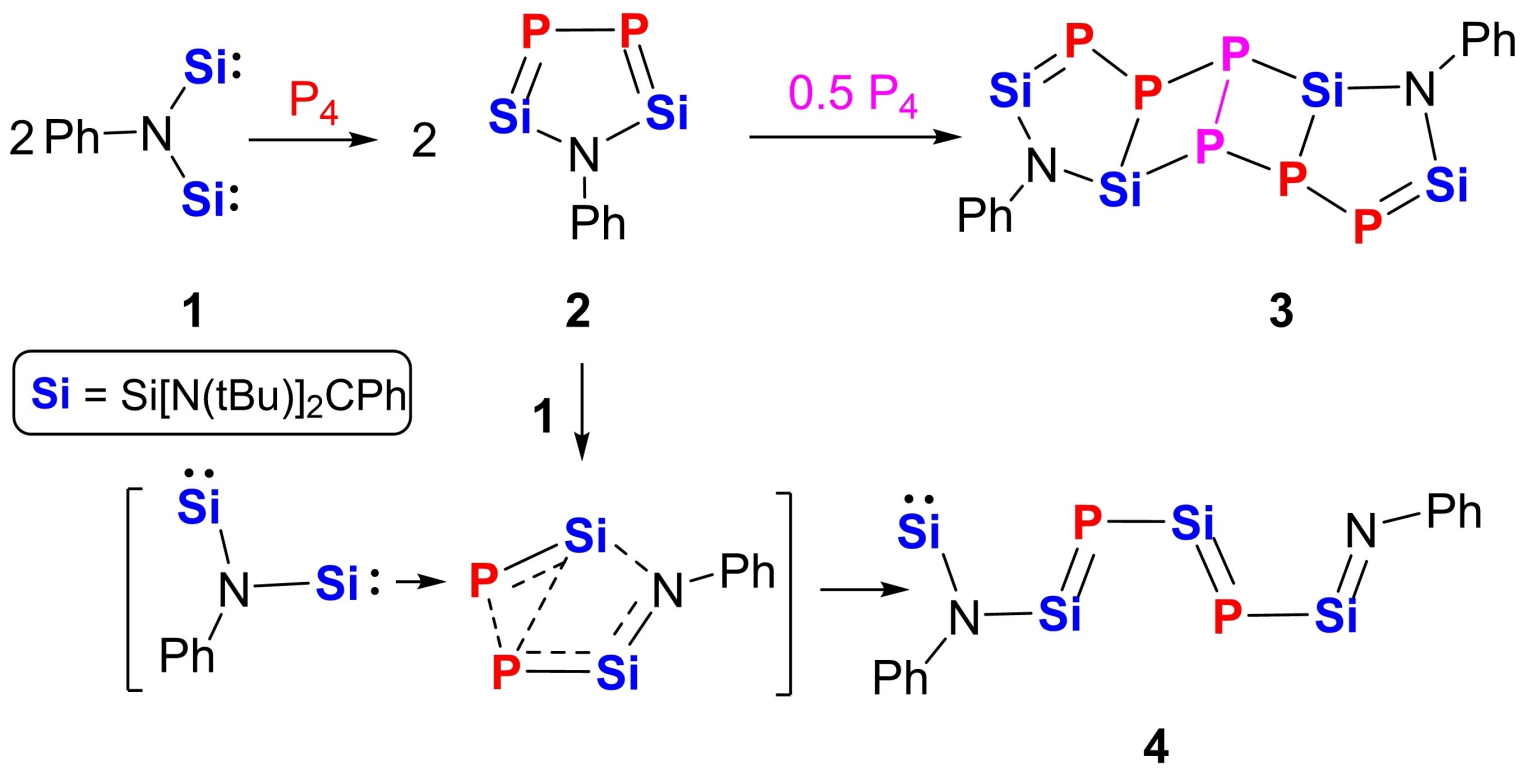
**2** as a reaction intermediate in the formation of **3** and **4**.

Implied by the formation of a P_2_ adduct of the bis(silylene) (Xant)_2_ **Si**_2_P_2_ **B**,^[10]^ the related P_2_ species PhN**Si**_2_P_2_ **2** supported by PhN**Si**_2_ **1** was proposed to be an intermediate in the reaction of **1** and P_4_, which reacts probably spontaneously with P_4_ and **1** to yield **3** and **4**, respectively. In order to catch the proposed intermediate **2**, compound **1** was treated with P_4_ in a molar ratio of 2 : 1 at low temperature (−60–−20 °C). To our delight, the formation of PhN**Si**_2_P_2_ **2** at −20 °C could be confirmed by ^31^P{^1^H}, ^29^Si{^1^H} and ^1^H NMR spectra. In fact, **2** was isolated in 17 % yields in the form of black crystals (Scheme 3). Moreover, starting from the isolated pure compound **2**, its reaction with P_4_ and **1** afforded **3** and **4**, respectively (Scheme 4). The high reactivity of **2** toward both P_4_ and **1** differs significantly from **B**, which is inert in the presence of P_4_ or (Xant)**Si_2_** and was even isolated at room temperature in high yields (91 %).^[10]^

As shown in Scheme 4, although the reaction mechanism for the reaction of **2** with **1** is unknown, it is reasonable to assume that the initial step should be the nucleophilic attack of a silylene moiety in **1** to one of the P atoms in **2**, which triggered the cleavage of the P−P bond in **2** and formation of two new Si=P bonds to afford **4** under cleavage of a Si−NPh bond. On the other hand, starting from **2**, its reaction with P_4_ may proceed via cycloaddition of one Si=P bond in **2** with a P_2_ unit of P_4_ to yield **3**.

### Characterization and Electronic Structure of 2

**Figure 2:**
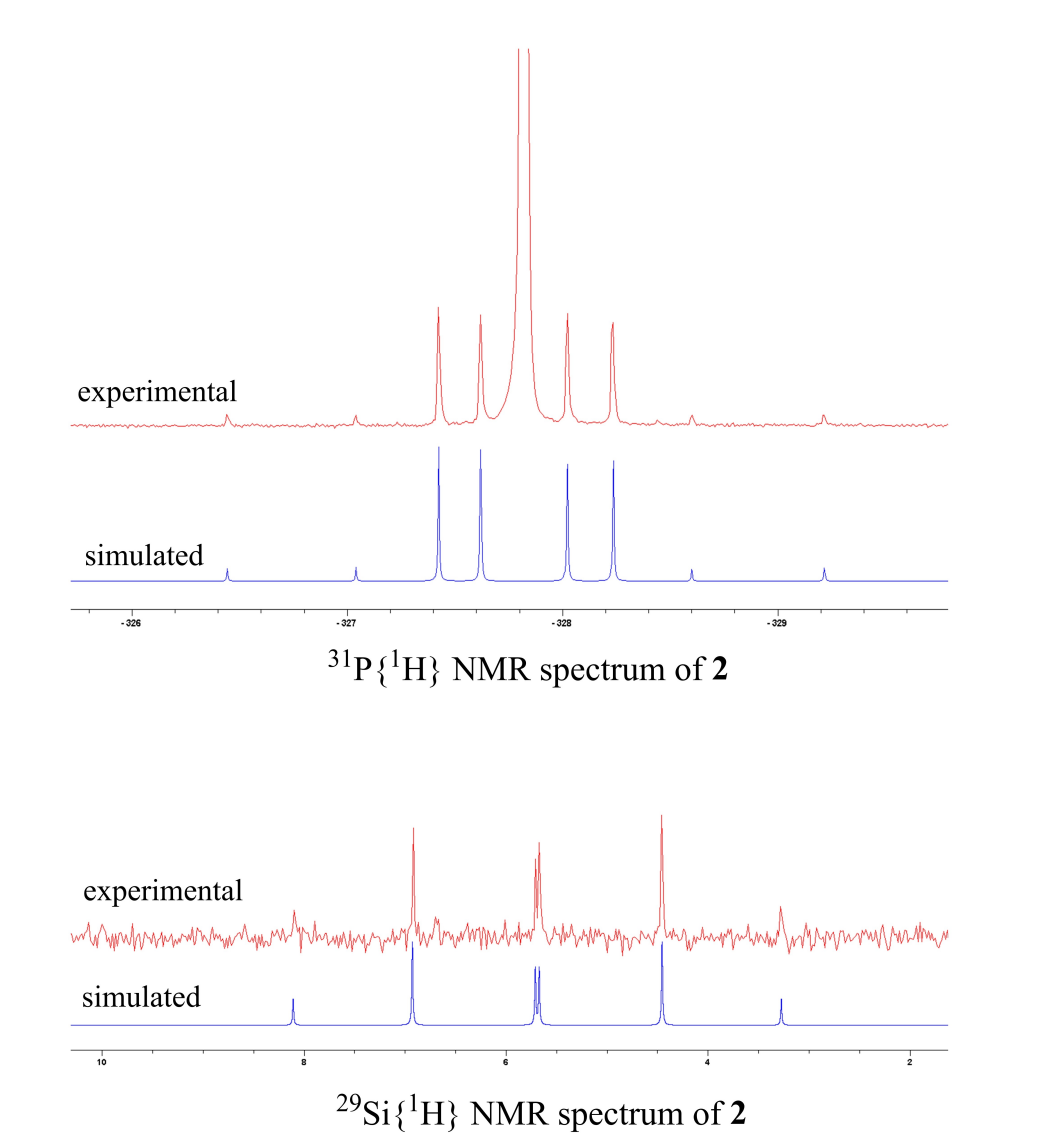
Experimental ^31^P{^1^H} NMR spectrum (161.98 MHz, THF‐*d*_8_/C_6_D_6_ 298 K) and simulated spectrum of **2** (^29^Si isotopologue) (top). Experimental ^29^Si{^1^H} NMR spectrum (79.49 MHz, THF‐*d*_8_/C_6_D_6_, 298 K) and simulated spectrum of **2** (bottom).

The ^1^H NMR spectrum of **2** exhibits one singlet for the four *t*Bu groups at *δ*=1.24 ppm, indicating the symmetrical structure of **2** in solution. Similarly, in the ^31^P{^1^H} NMR spectrum of **2**, only one signal at *δ*=−328.0 ppm (vs. −282.4 ppm in **B** ^[10]^) with ^29^Si satellites is observed (Figure 2). In line with that, the ^29^Si{^1^H} NMR spectrum of **2** shows a higher‐order spin‐system at *δ*=5.7 ppm (vs. 3.7 ppm in **B** ^[10]^) due to an ABX system resulting ^29^Si‐^31^P and ^31^P‐^31^P coupling (Figure 2). In order to figure out the coupling constants between the ^29^Si and ^31^P nuclei, a simulation was performed and the simulated ^31^P{^1^H}‐ and ^29^Si{^1^H} NMR spectra agree well with the respective experimental features and values (Figure 2, Figure S7, S9).

**Figure 3:**
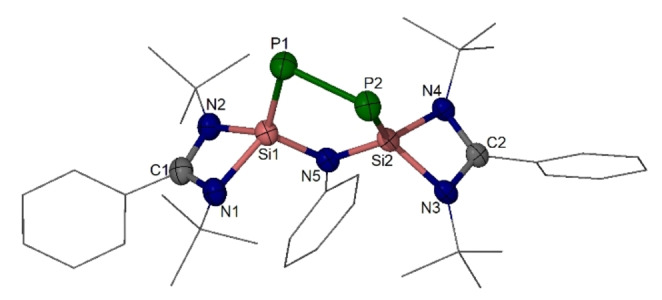
Molecular structure of **2**.^[20]^ Thermal ellipsoids are drawn at 50 % probability level. H atoms are omitted for clarity. Selected interatomic distances [Å] and angles [°]: Si2−N5 1.736(1), Si1−N5 1.749(1), Si2−P2 2.115(1), Si1−P1 2.109(1), P2−P1 2.265(1); N5−Si1−P1 113.8(1), N5−Si2−P2 112.1(1), Si1−P1−P2 92.4(1), Si2−P2−P1 95.5(1), Si2−N5−Si1 109.6(1).

Compound **2** is stable at room temperature both in solution and in the solid state under an inert atmosphere. In THF, **2** crystallizes in the monoclinic space group *P*2_1_/*c* (Figure 3). Its molecular structure was established by XRD, revealing a five‐membered NSi_2_P_2_ ring in a strongly puckered shape. The P−P distance of 2.265(1) Å in **2** represents a P−P single bond, which is slightly shorter than that in **B** (2.237(1) Å).^[10]^ The average Si−P distance of 2.130(1) Å in **2** is close to that in **B** (2.130(1) Å)^[10]^ and **D** (2.118(1) Å),^[6]^ indicating double bond character for the Si−P bonds in **2**.

**Figure 4:**
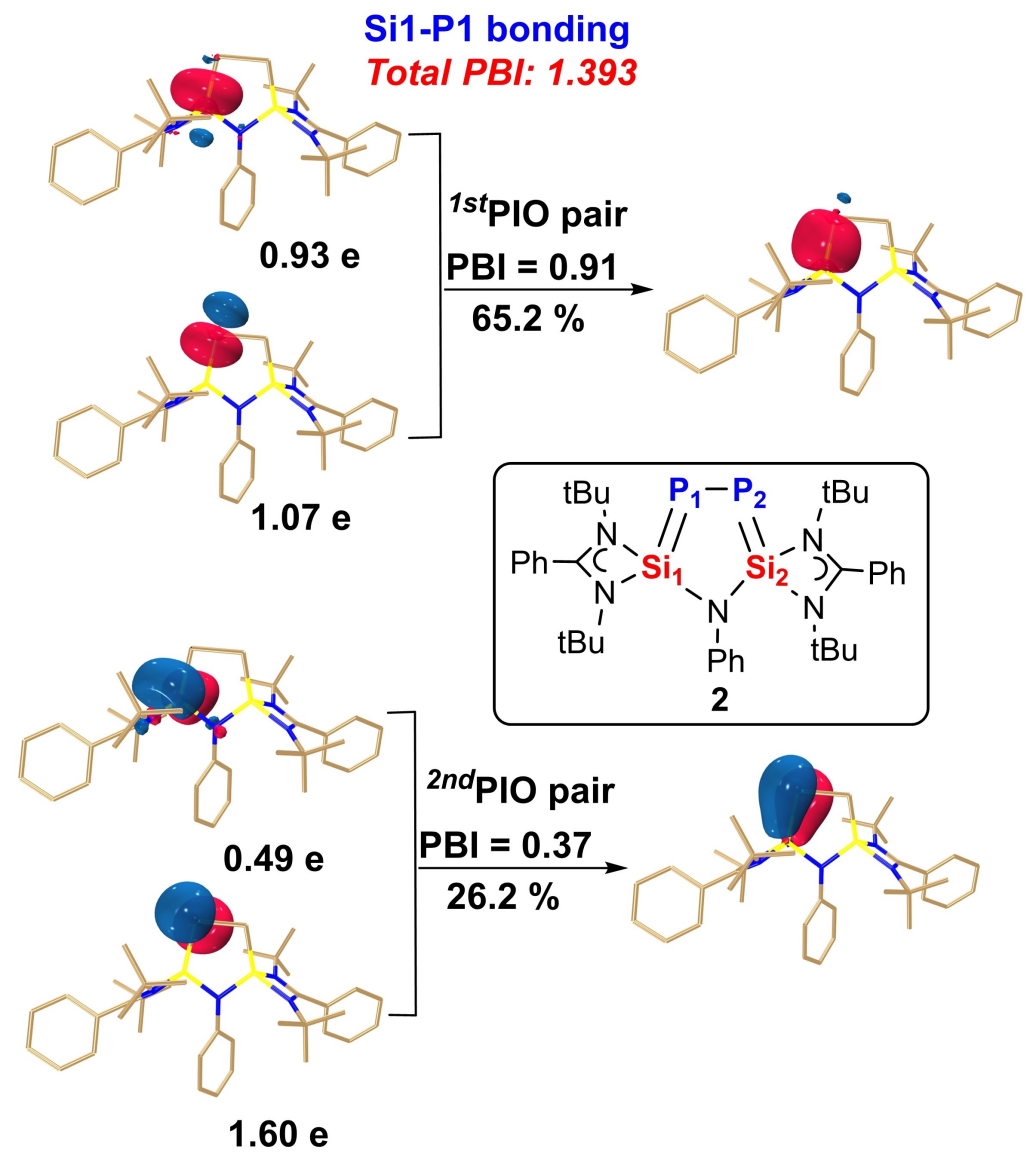
Principal Interacting Orbital (PIO) analysis on the bonding modes between Si1‐P1 atoms in compound **2**. Hydrogen atoms in 3D structures are omitted for clarity. Each PIO pair leads to a bonding PIMO (principal interacting molecular orbital). The PIO‐based bond index (PBI) can quantify the strength of the interaction. The PIO analysis is performed by cutting the Si−P bond; the isosurface 0.050 au is plotted.

DFT calculations confirmed the XRD structure of **2**. The dominant interaction of Si−P and P−P bonds in **2** was investigated with the Principal Interacting Orbital (PIO) analysis developed by Lin[^16^, ^17^] (Figure 4, Figure S26). For the Si1−P1 bonding, two dominant contributing PIO pairs with PBIs of 0.91 and 0.37, respectively, as well as the total PBI of 1.393, were located (Figure 4), suggesting a formal double bond character between Si1 and P1 atoms. Specifically, the first and second PIO pairs suggest a σ‐bonding and a π‐bonding interaction between the Si1 and the P1 atoms. Note that the analysis of Si2‐P2 bond showed an identical result (Figure S26b). Thus, the two Si−P bonds in **2** exhibit Si=P bond character, as revealed by XRD, which is also supported by Wiberg bond index (WBI) analysis based on Natural Bond Orbital (NBO) analysis (Figure S25a). On the other hand, the only one dominant contributing PIO pair with PBI of 0.87 and the total PBI of 0.917 (Figure S26c) indicated a P−P single bond in **2**, which agrees also well with the WBI of 0.917 (Figure S25a).

Compound **2** represents the first isolable 2,5‐disila‐3,4‐diphosphapyrrole derivative. To further explore the degree of conjugation of the Si=P bonds within the five‐membered P_2_Si_2_N ring in **2**, the electron density of delocalized bonds (EDDB) was employed. For comparison, the parent pyrrole was also investigated (Figure S27). Both the π‐electron delocalization of C=C−C=C and the five‐membered C_4_N ring in the parent pyrrole is significantly stronger than those of Si=P−P=Si and the five‐membered Si_2_P_2_N ring in **2** (Figure S27). As expected, the C−C distances in pyrrole are more equalized, whereas the P−P bond in **2** suffers from electrostatic repulsion, due to the pronounced zwitterionic character of the Si=P (Si^+^−P^−^) bonds, leading to a somewhat longer P−P distance (2.265(1) Å) in a strongly puckered ring. This striking break of delocalization in **2** is also reflected by nucleus‐independent chemical shift [NICS(1)zz] calculations. The respective results are shown in Figure S28b (−31.7 ppm for pyrrole vs. +4.4 ppm for **2**), indicating a non‐aromatic character of **2** compared to aromatic pyrrole.

According to DFT calculations, the HOMO in **2** is mainly localized on the five‐membered Si_2_P_2_N‐ring moiety, whereas the LUMO is mostly localized on the phenyl rings of both amidinate ligands (Figure S28). The maximum UV/Vis absorption wavelength of **2** at *λ*_exp_=568 nm (vs. cal. 600.2 nm) can be assigned to the electronic transitions of HOMO→LUMO+2 (19.2 %) and HOMO→LUMO+5 (72.2 %). In addition, an absorption shoulder at 426 nm (vs. cal. 421.2 nm) can be assigned to the electronic transition of HOMO‐1→LUMO (77.8 %) (Figure S29).

### Interconversion between 3 and 4

**Scheme 5:**
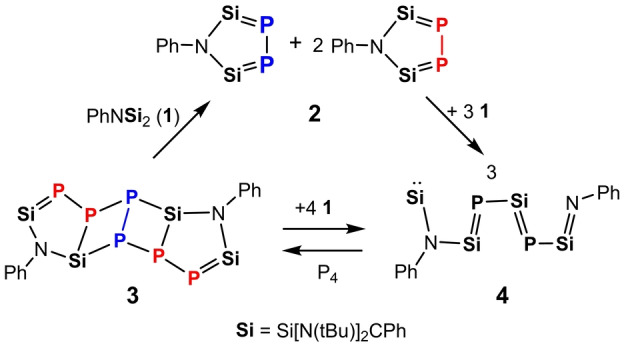
Interconversion of **3** and **4**.

Featuring intact P−P bonds in the molecule, compound [PhN**Si**_2_P_2_]_2_P_2_ **3** could be further activated when treated with silylenes. In fact, compound **3** consumes four molar equivalents of the bis(silylene) **1** to afford **4** (Scheme 5). Unexpectedly, the latter reaction proceeds via formation of **2** as proven by ^31^P NMR spectroscopy (Figure S18). Presumably, the P_2_ unit in the middle of the molecule **3** is extracted by **1** to form **2** along with release of two molecules of **2** from **3** (Scheme 5). The same P_2_ transfer reaction mode was confirmed by the reaction of **3** with (Xant)**Si**_2_,^[11]^ which yielded a reaction mixture with a signal at *δ*=−282.3 ppm for **B** and at −328.0 ppm for **2** in the ^31^P{^1^H} NMR spectrum (Figure S19). On the other hand, possessing a Si=P and Si=N conjugated chain functionalized with a silylene moiety, compound **4** may be able to activate P_4_. Thus **4** was treated with equimolar amounts of P_4_. Unexpectedly, the ^31^P{^1^H} NMR spectrum of the reaction mixture exhibited a fast conversion of **4** to **3** (Scheme 5). We reason that the initial step could be the nucleophilic attack of the terminal Si^II^ atom in **4** to one of the P atoms in P_4_.[^6^, ^18^] Notably, the terminal PhN=Si moiety of **4** is transformed to a Si−N(Ph)−Si sequence again during the reaction process.

### Characterization and Electronic Structure of 3

**Figure 5:**
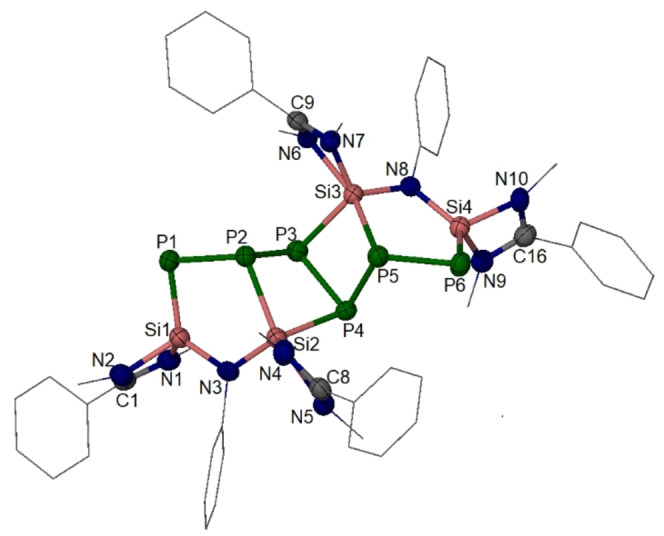
Molecular structure of **3**.^[20]^ Thermal ellipsoids are drawn at 50 % probability level. H atoms and all Me groups from *t*Bu groups are omitted for clarity. Selected interatomic distances [Å] and angles [°]: P6−P5 2.192(1), P5−P4 2.249(2), P4−P3 2.218(2), P3−P2 2.234(2), P2−P1 2.192(1), Si3−P5 2.330(2), Si3−P3 2.275(1), Si4−P6 2.121(2), Si2−P4 2.273(2), Si2−P2 2.335(2), Si1−P1 2.119(2), P6−P5−P4 105.5(1), P5−P4−Si2 100.1(1), Si2−N3−Si1 119.0(2), Si4−N8−Si3 117.8(2), N8−Si3−P3 116.8(1), P1−P2−P3 105.2(1), N3−Si2−P4 120.8(1), P2−Si2−N5 164.7(1), P5−Si3−N6 163.5(1).

The ^1^H NMR spectrum of **3** exhibits four singlets at *δ*=0.87, 0.92, 1.50, and 1.65 ppm for the eight *t*Bu environments. Accordingly, the ^31^P{^1^H} NMR spectrum of **3** shows only three resonances for P1(P6) (*δ*=−247.0 ppm), P3(P4) (*δ*=−34.2 ppm), and P2(P5) atoms (*δ*=40.2 ppm) (Figure 5). The resonance at the highest field for Si=*P*1(*P*6) at *δ*=−247.0 ppm in **3** exhibits a slightly down‐field shift compared with those in **B** (*δ*=−282.4 ppm),^[10]^ **D** (*δ*=−269.8 ppm),^[15]^ and **2** (*δ*=−328.0 ppm). This assigment is supported by theoretical calculations of the ^31^P NMR chemical shifts for **3** (*δ*=−235.1 ppm for P1(P6), −26.7 ppm for P3(P4), and 41.5 ppm for P2(P5) (Figure S30). Furthermore, in the ^29^Si{^1^H} NMR spectrum of **3**, the resonance at *δ*=7.2 (vs. *δ*=5.7 ppm in **2** and *δ*=3.7 ppm in **B**)^[10]^ can be assigned to the four coordinate Si1 atom (Figure 5), whereas the signal at *δ*=−57.5 ppm can be assigned to the five coordinate Si2 atom. The latter agrees with the calculated values (*δ*=−1.6 ppm for Si1, and −70.4 ppm for Si2 (Figure S31).

Compound **3** crystallizes in THF in the triclinic space group *P*‐1. (Figure 5). The core of tetracyclic **3** consists of two five‐membered NSi_2_P_2_ rings and two four‐membered SiP_3_ rings. All these rings are slightly puckered. A C2 axis passes perpendicularly through the P3−P4 bond center in **3**, accounting for the aforementioned NMR spectroscopic data. The Si1 and Si4 atoms adopt a four‐coordinate tetrahedral geometry with one P atom and three N atoms from the ligand backbone. On the other hand, the Si2 and Si3 atoms are five‐coordinate with two P atoms and three N atoms from the ligand backbone, displaying a distorted trigonal‐bipyramidal coordination geometry, in which the P2 and N5 atoms are located in the apical positions with P2−Si2−N5 angles of 164.7(1)°. The averaged Si=P bond length of 2.120(1) Å in **3** is close to those values in **2** (2.130(1) Å), **B** (2.130(1) Å),^[10]^ and **D** (2.130(1) Å),^[6]^ but shorter than that in **A** (2.195(1) Å).^[9a]^ The average Si−P single bond lengths of Si2−P2_apical_ 2.333(2) Å and Si2−P4_equatorial_ (2.274(1) Å) in **3** are in the common range of Si−P single bonds.[^6^, ^7^, ^8^, ^19^]

**Figure 6:**
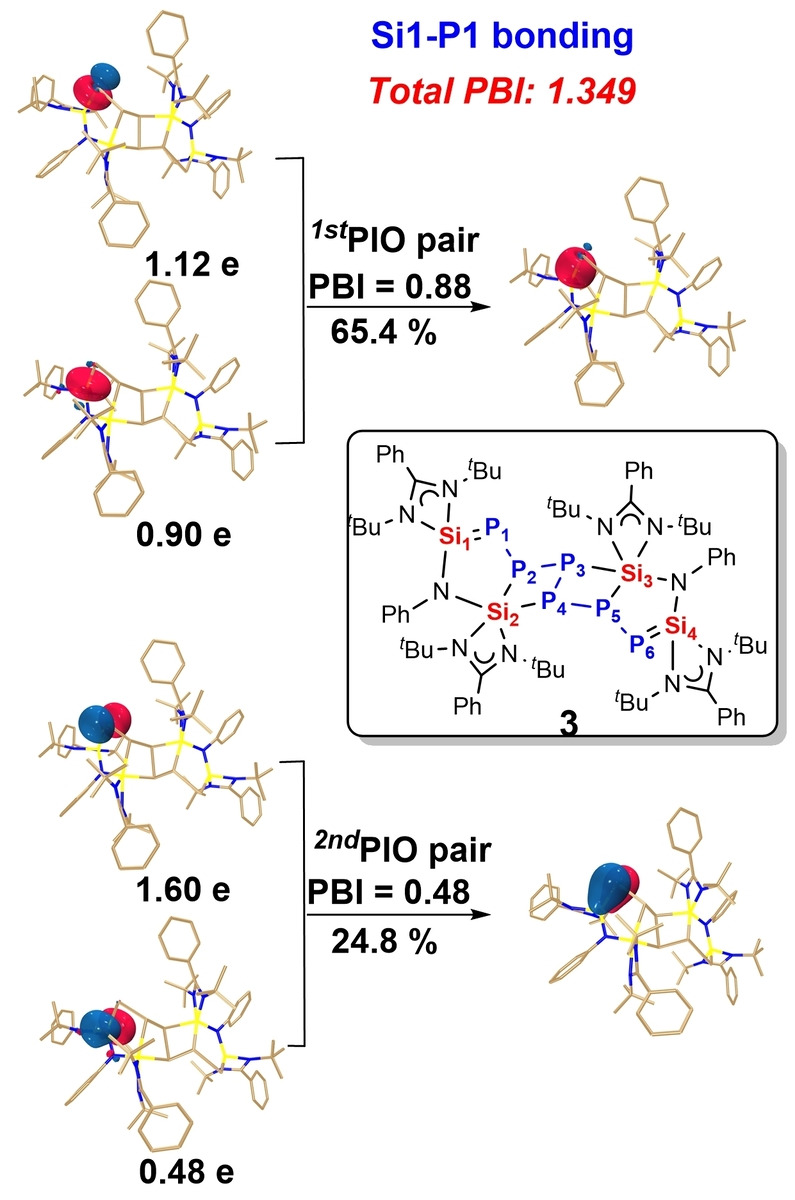
PIO analysis on the bonding modes of Si1‐P1 in compound **3**. Hydrogen atoms in 3D structures are omitted for clarity. The isosurface 0.050 au is plotted.

DFT calculations[^16^, ^17^] confirmed the metric parameters of **3** (Figures S33–S35). For the Si1−P1 bond, the PIO pairs with PBIs of 0.88 and 0.48, respectively (Figure 6), as well as the WBI of 1.349, indicate Si1=P1 bond properties in **3**. In addition, both of the PIO analysis (Figure S33–35) and WBI (Figure S32) confirm the *σ* type Si2−P2 and Si2−P4 bonds.

### Characterization and Electronic Structure of 4

**Figure 7:**
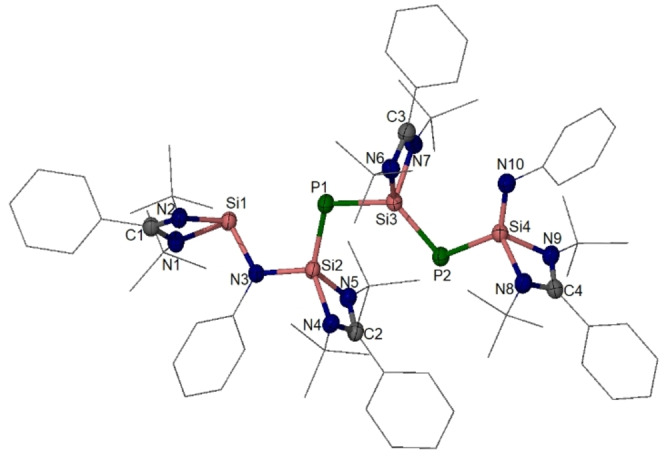
Molecular structure of **4**.^[20]^ Thermal ellipsoids are drawn at 50 % probability level. H atoms are omitted for clarity. Selected interatomic distances [Å] and angles [°]: Si1−N3 1.803(1), N3−Si2 1.728(2), Si2−P1 2.127(1), P1−Si3 2.186(1), Si3−P2 2.145(1), P2−Si4 2.178(1), Si4−N10 1.625(2); Si3−P2−Si4 104.1(1), N3−Si2−P1 108.6(1), P2−Si3−P1 123.9(1), Si2−P1−Si3 108.7(1), N10−Si4−P2 125.8(1), Si2−N3−Si1 115.7(1).

Owing to the symmetrical orientation of the two *t*Bu groups in each **Si** moiety in **4**, the eight *t*Bu groups in **4** give rise to only four singlets in the ^1^H NMR spectrum at *δ*(ppm) 1.13 (*s*, 18 H), 1.28 (*s*, 18 H), 1.35 (*s*, 18 H), and 1.51 (*s*, 18 H). Similarily, the ^31^P{^1^H} NMR spectrum of **4** shows only two doublets at *δ* (ppm) −263.8 (P1, see Figure 7) and −287.6 (*P2*), which correlate well with the calculated chemical shifts (*δ*=−263.9 ppm for P1 and −293.2 ppm for P2, Figure S37). These strong up‐field shifts are consistent with a zwitterionic Si=P bond character of **4**, and comparable with those in **B** (*δ*=−282.4 ppm),^[10]^ **D** (*δ*=−269.8 ppm),^[6]^ **3** (*δ*=−247.0 ppm), and **2** (*δ*=−328.0 ppm). In the ^29^Si NMR spectrum of **4**, four sets of resonances at *δ* (ppm) 46.2 (Si3, see Figure 7), 9.9 (Si2), −27.4 (Si1), and −31.6 (Si4) are in line with the results from DFT calculations (*δ*=46.7 ppm, Si3; 12.1 ppm, Si2; −19.6 ppm, Si1; and −29.3 ppm, Si4. Figure S38), in which the two signals at the low field region for Si3 (*δ*=46.7 ppm) and Si2 (*δ*=12.1 ppm) from Si=P moieties are comparable with those in **A** (*δ*=26.5 ppm),^[9a]^ **B** (*δ*=3.7 ppm),^[10]^ **D** (*δ*=50.2, 33.9 ppm),^[6]^ **2** (*δ*=5.7 ppm), and **3** (*δ*=7.2 ppm), indicating the zwitterionic character of the P2=Si3 and P1=Si2 bonds in **4**.

Compound **4** crystallizes in Et_2_O in the triclinic space group *P*‐1. Its XRD analysis revealed a chain‐shaped molecule containing a three‐coordinate silylene‐silicon atom (Si1) in a typical trigonal‐pyramidal coordination environment (Figure 7). The other three Si atoms (Si2, Si3, Si4) are four‐coordinated, each adopting a distorted tetrahedral coordination geometry. Two short Si−P (Si2−P1: 2.127(1) and P2−Si3: 2.145(1) Å) and two long Si−P distances (Si3−P1: 2.186(1) and P2‐Si4: 2.178(1) Å) are observed in **4**. Together with the relatively short Si4−N10 bond (1.625(2) Å), the geometrical parameters of **4** indicate a π‐conjugated :Si1−N3−Si2=P1−Si3=P2−Si4=N10 chain located in one slightly puckered plane.

**Figure 8:**
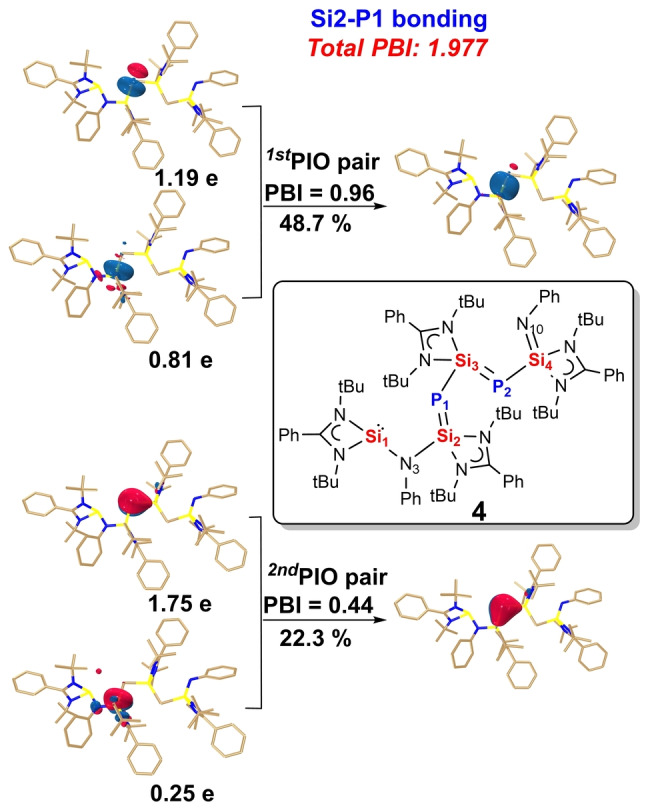
PIO analysis on the bonding modes of Si2‐P1 bond in compound **4**. Hydrogen atoms in 3D structures are omitted for clarity. The PIO analysis is performed by cutting the Si2‐P1 bond. The isosurface 0.050 au is plotted.

DFT calculations with the PIO and WBI analyses[^16^, ^17^] confirmed the π‐delocalized structure (Figure 8) for Si2−P1 bond, Figure S41–S43 for all other Si−P bonds, Figure S39 for WBI). The total principal bond index (PBI) values of 1.98 for Si2−P1 (Figure 8) and 1.81 for Si3−P2 bond (Figure S42) indicate their double bond characters. In contrast, the total PBI values of 1.63 for Si3‐P1 (Figure S41) and 1.56 for Si4−P2 bond (Figure S43) indicate a significant conjugation. Specifically, the first PIO pair with different PBI values (0.95–0.96) suggests *σ* type donation from the lone pair electrons of the Si atom to the P atom, while the second PIO pair with different PBI values (0.28–0.44) illustrates π‐backdonation of a lone pair of electrons on the P atom to the vacant *p* orbital of the Si atom. Their difference of the second PIO pair leads to the different bond strength, which might depend on the electron‐donating ability of the corresponding P atoms.

## Conclusion

In summary, the first isolable non‐aromatic 2,5‐disila‐3,4‐diphosphapyrrole PhN**Si**_2_P_2_ **2**, the polycyclic phosphasilene [PhN**Si**_2_P_2_]_2_P_2_ **3**, and silylene‐phosphasilene **4** featuring the π‐conjugated :**Si**−N(Ph)−**Si**=P−**Si**=P−**Si**=NPh chain are obtained from white phosphorus activation with the new bis(silylene) PhN**Si_2_ 1**. Due to the relatively short Si^II^−Si^II^ distance of 2.895(1) Å in **1**, the outcome of the metal‐free, cooperative bis(silylene)‐assisted P_4_ activation modes is unique and different from that of other bis(silylenes) with greater Si^II^−Si^II^ distances and subtly different electronic profiles. Compounds **3** and **4** can convert into each other via reactions with **1** and P_4_, respectively. DFT calculations including PIO and NBO analyses for **2**, **3**, and **4** were investigated, which agree well with the respective experimental results. Compounds **2**, **3**, and **4** are promising electron‐rich Si=P ligands to generate polydentate phosphasilene‐ and disiladiphosphapyrrole‐metal π‐complexes. Moreover, **3** may serve as a P_2_‐transfer reagent for other silylene analogues. Respective investigations are in progress.

## Conflict of interest

The authors declare no conflict of interest.

1

## Supporting information

As a service to our authors and readers, this journal provides supporting information supplied by the authors. Such materials are peer reviewed and may be re‐organized for online delivery, but are not copy‐edited or typeset. Technical support issues arising from supporting information (other than missing files) should be addressed to the authors.

Supporting Information

## Data Availability

The data that support the findings of this study are available in the Supporting Information of this article.
