# Supplementary material for: An Isolable 2,5‐Disila‐3,4‐Diphosphapyrrole and a Conjugated Si=P−Si=P−Si=N Chain Through Degradation of White Phosphorus with a N,N‐Bis(Silylenyl)Aniline
Source: Angew Chem Int Ed Engl. 2022 Aug 8;61(37):e202209250. doi: 10.1002/anie.202209250 (PMC9545316; doi:10.1002/anie.202209250)
Supplement: Supplementary file 1 — Supporting Information [file ANIE-61-0-s001.pdf]

Angewandte Chemie
Eine Zeitschrift der Gesellschaft Deutscher Chemiker

Supporting Information

An Isolable 2,5-Disila-3,4-Diphosphapyrrole and a Conjugated Si=P–Si=P–Si=N Chain Through Degradation of White Phosphorus with a *N*,*N*-Bis(Silylenyl)Aniline

*Y. Xiong, S. Dong, S. Yao, C. Dai, J. Zhu, S. Kemper, M. Driess**

Table of Content

A Experimental Section……………………………………………………………………………3

A1 General considerations……………………………………………………………………………3

A2 Single-crystal X-ray structure determination……………………………………………………3

A3 Synthesis, characterization and reactivity…………………………………………………………3

A3.1 Synthesis and characterization of **1 - 4**……………………………………………………3

A3.2 Reactivity of **2**, **3** and **4**…………………………………………………………………15

A4 Details of the single crystal X-ray diffraction analyses…………………………………………17

B Computational Section……………………………………………………………………………28

C References………………………………………………………………………………………46

A. Experimental Section

A1. General considerations

All experiments and manipulations were carried out under dry nitrogen using standard Schlenk techniques or in an MBraun inert atmosphere dry box containing an atmosphere of purified N_2. Solvents were deoxygenated and dried by standard methods, saturated with purified N_2 and freshly distilled prior to use. The precursor compounds $PhNLi_2$ [1] and PhC[(*t*Bu)N]$_2$SiCl [2] were prepared according to literature procedure. The ^{1}H, ^{13}C, ^{31}P, ^{29}Si-NMR spectra were recorded on Brucker AV200, AV400, AV500 spectrometers referenced to residual solvent signals as internal standards (^{1}H NMR: C_6D_6, 7.16 ppm; THF-d_8, 1.76 and 3.62 ppm; $^{13}C\{^{1}H\}$ NMR: C_6D_6, 128.06 ppm; THF-d_8, 26.19 and 68.26 ppm) or an external standard (^{29}Si NMR: $SiMe_4$, 0.0 ppm; ^{31}P NMR: 85% H_3PO_4, 0.0 ppm). ^{1}H NMR spectrum of **2** was calibrated using TMS as external standard, the other nuclei (^{13}C, ^{29}Si and ^{31}P) of this compound were calibrated using the unified scale. Abbreviations: *s* = singlet; *d* = doublet; *t* = triplet; *sept* = septet; *m* = multiplet; *br* = broad; *dd* = doublet of doublets, *dt* = doublet of triplets and so on. IR spectra were measured with a Nicolet iS5 FT-IR Spectrometer from the company of Thermo Scientific. UV/Vis spectra were recorded on an Analytik Jena Specord S600 diode array spectrometer. Elemental analyses were performed on a Flash EA 1112 CHNS Analyzer. Melting points were measured on a Stuart SMP30 melting point apparatus.

A2. Single-crystal X-ray structure determinations

Crystals were each mounted on a glass capillary in per-fluorinated oil and measured in a cold N_2 flow. The data of **1-4** were collected on an Oxford Diffraction Supernova, Single source at offset, Atlas at 150 K (Cu- Kα-radiation, λ = 1.5418 Å). The structures were solved by direct method and refined on F2 with the SHELX-97 [3] software package. The positions of the H atoms were calculated and considered isotropically according to a riding model. Several co-crystallized free THF molecules in **2** and **3**, Et_2O in **4** within the asymmetric unit are highly disordered and treated using the SQUEEZE routine in PLATON. CCDC 2175471 (**1**), CCDC 2175474 (**2**), CCDC 2175473 (**3**), and CCDC 2175472 (**4**) contain the supplementary crystallographic data for this paper. These data can be obtained free of charge from The Cambridge Crystallographic Data Centre via www.ccdc.cam.ac.uk./data_request/cif.

A3. Synthesis, characterization and reactivity of 1 - 4

A3.1 Synthesis and characterization of 1 - 4

Compound 1: To a 250 mL flask with 100 mL Et_2O solution of PhC[(*t*Bu)N]$_2$SiCl (6.50 g, 22.0 mmol) at −30 °C was added slowly $PhNLi_2$ (1.16 g, 11.0 mmol) under stirring. The reaction mixture was allowed to warm to ambient temperature slowly and stirring for 4 h. A brown solution and precipitate (containing **1** and LiCl) were formed. Compound **1** was extracted with Et_2O (3x50 mL) from the isolated precipitate. The concentrated extraction yielded at 4 °C yellow crystals of **1** (4.37 g, 7.15 mmol, 65 % yield). M.p. 181 ^{o}C (decomp.); **^{1}H NMR** (200.13 MHz,

THF-d_8, 298 K): δ (ppm) = 1.04 (*s*, 36 H; Si-NC(C*H*$_3$)$_3$), 6.83 – 7.48 (*m*, 15 H; *Ph*); **^{13}C{^{1}H} NMR** (50.32 MHz, THF-d_8, 298 K): *δ* (ppm) = 32.65 (*C*H$_3$)$_3$), 54.22 (*C*(CH$_3$)$_3$), 121.88, 125.32, 128.84, 129.10, 129.36, 130.06, 130.96 132.28 (*Ph*), 136,33, 151.34 (quaternary *Ph*), 162.93 (NCN); **^{29}Si{^{1}H} NMR** (79.49 MHz, THF-d_8, 298 K): δ(ppm) = -12.9 ppm (*s*); Elemental analysis calcd (%) for $C_{36}H_{51}N_5Si_2$ (610.0 g/mol) C 70.88, H 8.43, N 11.48, found: C 70.43, H 8.30, N 11.60. **IR** (cm^{-1}): 2959(*m*), 1587(*m*), 1479(*m*), 1443(*w*), 1410(*vs*), 1390 (*s*), 1359(*s*), 1267(*m*), 1214(*vs*), 1166(*m*), 1070(*m*), 1021(*m*), 1000(*m*), 927(*m*), 898(*s*), 833(*vs*), 827(*vs*), 794(*m*), 749(*s*), 738(*vs*), 709(*vs*), 697(*vs*), 659(*m*), 615(*m*), 604(*m*).

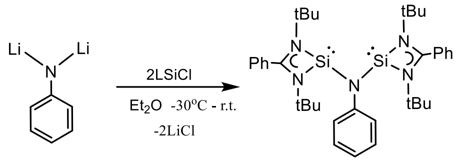

1 (65%)

Scheme S1. Synthesis of **1**

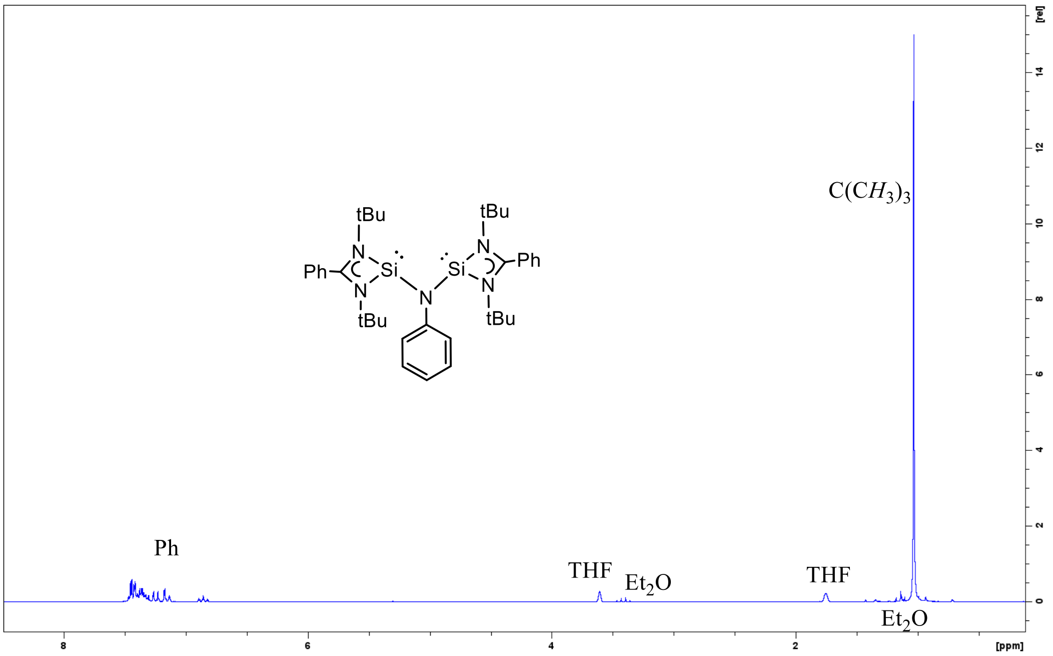

Figure S1. ^{1}H NMR spectrum (200.13 MHz, d_8-THF, 298 K) of **1**.

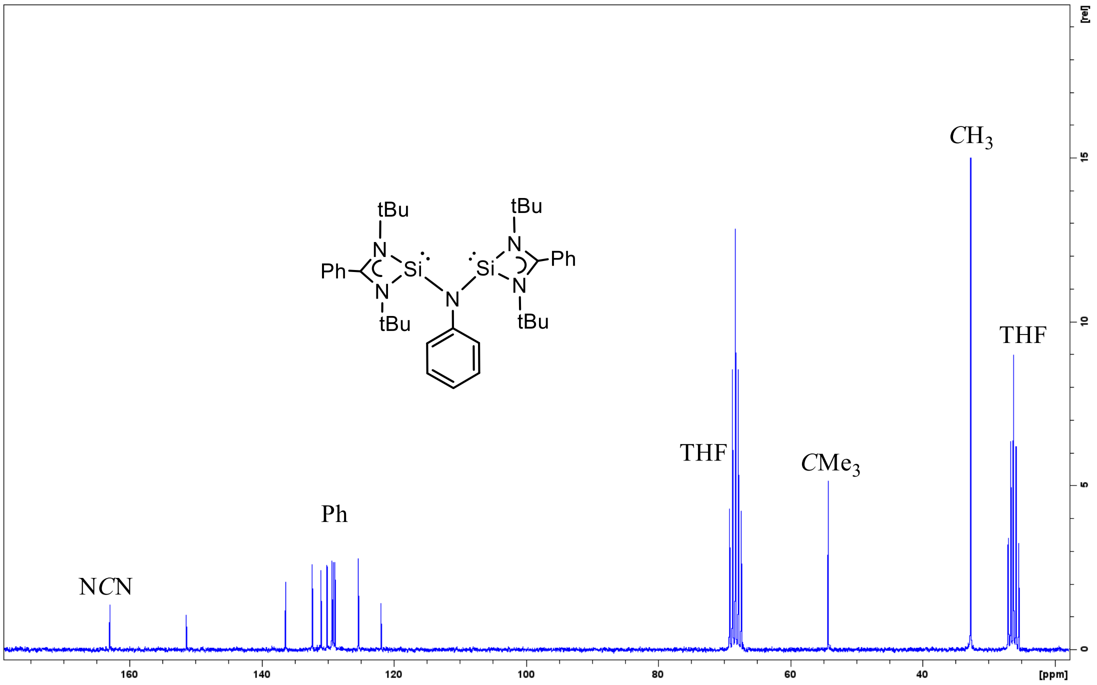

Figure S2. $^{13}C\{^{1}H\}$ NMR spectrum (50.32 MHz, d_8-THF, 298 K) of **1**.

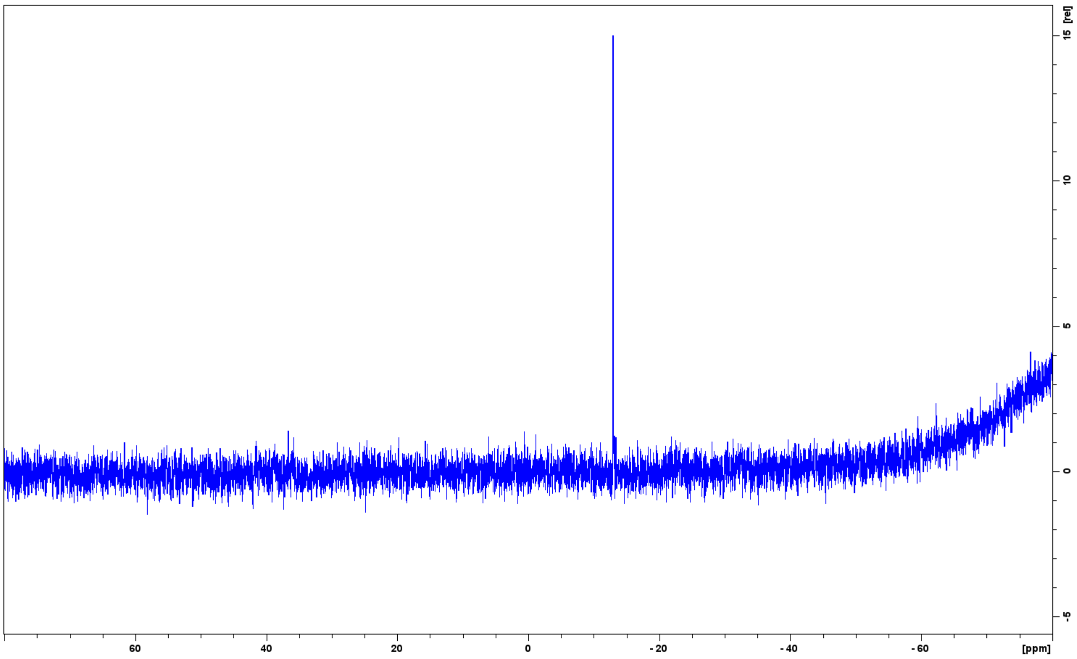

Figure S3. $^{29}Si\{^{1}H\}$ NMR spectrum (79.49 MHz, d_8-THF, 298 K) of **1**.

Compound 2: A colorless solution of P_4 (0.11g, 0.90 mmol) in 20 mL THF was added rapidly to a yellow solution of **1** (1.17 g, 1.92 mmol) in 50 mL THF at -20 °C under stirring. The solution turned to black immediately. The reaction mixture was stirred for one hour at that temperature. The volatiles were removed under reduced pressure. 10 mL Et_2O was added to the residue. From Et_2O solution at 4 °C black crystals of **2** (0.20 g, 0.30 mmol, 17% yield) were obtained. M.p. 206 °C (decomp.); **^{1}H NMR** (400.13 MHz, THF-d_8/C_6D_6, 298 K): δ(ppm) = 1.24 (s, 36 H, CH_3), 7.13 (*t*, 1H, J = 7.4 Hz), 7.29 (*d*, 4H, J = 7.4 Hz), 7.41 – 7.58 (*m*, 10H); **^{13}C{^{1}H} NMR** (100.61 MHz, THF-d_8/C_6D_6, 298 K): δ(ppm) = 33.01 (C(CH_3)$_3$), 55.98 (CMe$_3$), 124.12, 125.43, 129.51, 129.62, 129.91, 130.37, 130.42, 132.11(*Ph*), 132.88, 149.68 (quaternary *Ph*), 176.06 (N*C*N); **^{31}P{^{1}H} NMR** (161.98 MHz, THF-d_8/C_6D_6, 298 K): δ(ppm) = -328.0 (with Si satellite, non-first order spectrum); **^{29}Si{^{1}H} NMR** (79.49 MHz, THF-d_8/C_6D_6, 298 K): δ(ppm) = 5.7 (non-first order spectrum, $^1J_{PSi}$ = 206 Hz, $^2J_{PSi}$ = -9.5 Hz); Elemental analysis calcd (%) for $C_{36}H_{51}N_5Si_2P_2$: (671.95 g/mol) C 64.35, H 7.65, N 10.42, found: C 64.09, H 7.45, N 10.78. **IR** (cm^{-1}): 2969(*w*), 1592(*w*), 1494(*w*), 1474(*w*), 1447(*w*), 1401(*vs*), 1393(*vs*), 1363(*s*), 1279(*w*), 1248(*s*), 1202(*s*), 1090(*w*), 1024(*w*), 1001(*w*), 926(*vs*), 910(*w*), 866(*vs*), 796(*w*), 767(*vs*), 751 (*vs*), 705(*s*), 698(*s*), 684(*vs*), 661(*m*), 643(*vs*). UV-Vis: 426nm, ε = 1.5 x 10^5; 568 nm, ε = 9.6x10^4.

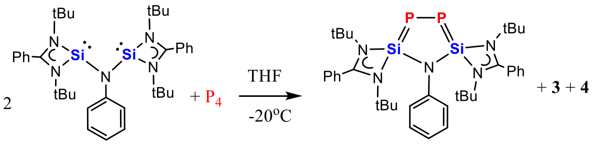

1 **2** (17%)

Scheme S2. Synthesis of **2**.

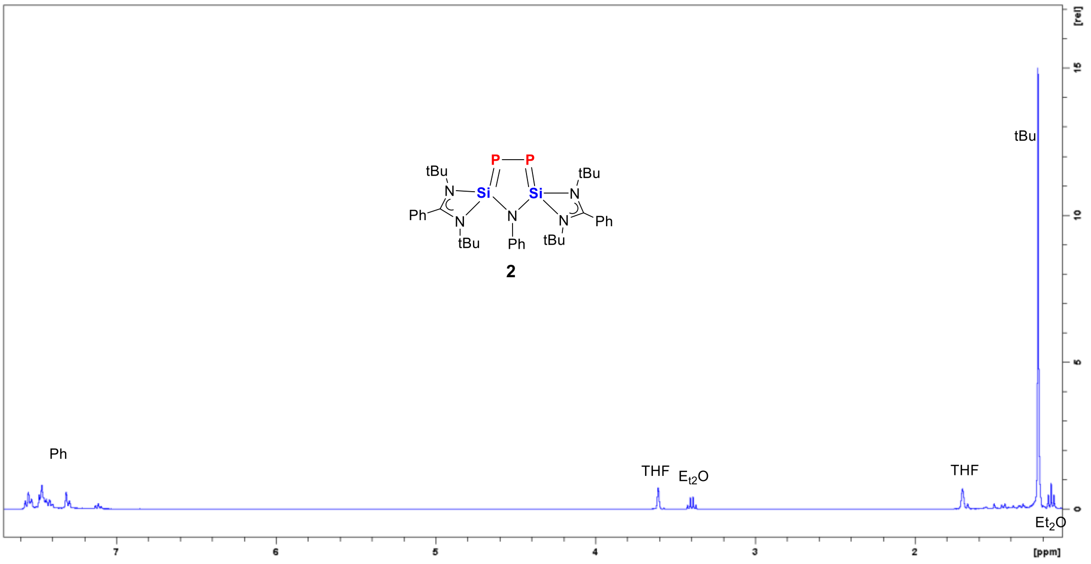

Figure S4. ^{1}H NMR spectrum (400.13 MHz, THF-d_8/C_6D_6, 298 K) of **2**.

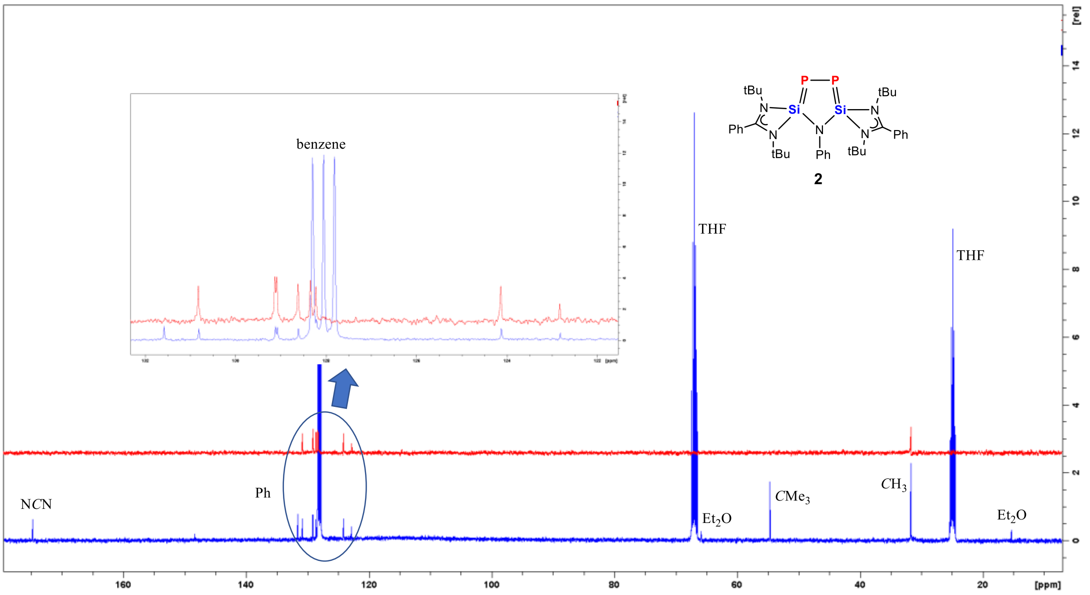

Figure S5. $^{13}C\{^{1}H\}$ (bottom) and DEPT-135 NMR (top) spectra (100.61 MHz, THF-d_8/C_6D_6, 298 K) of **2**.

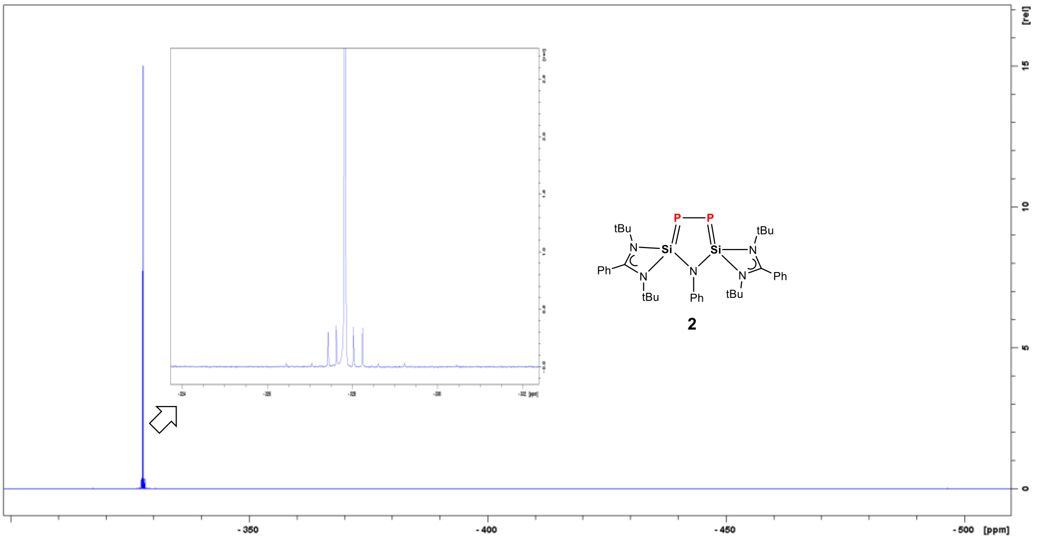

Figure S6. $^{31}P\{^{1}H\}$ NMR spectrum (161.98 MHz, THF-d_8/C_6D_6, 298 K) of **2**.

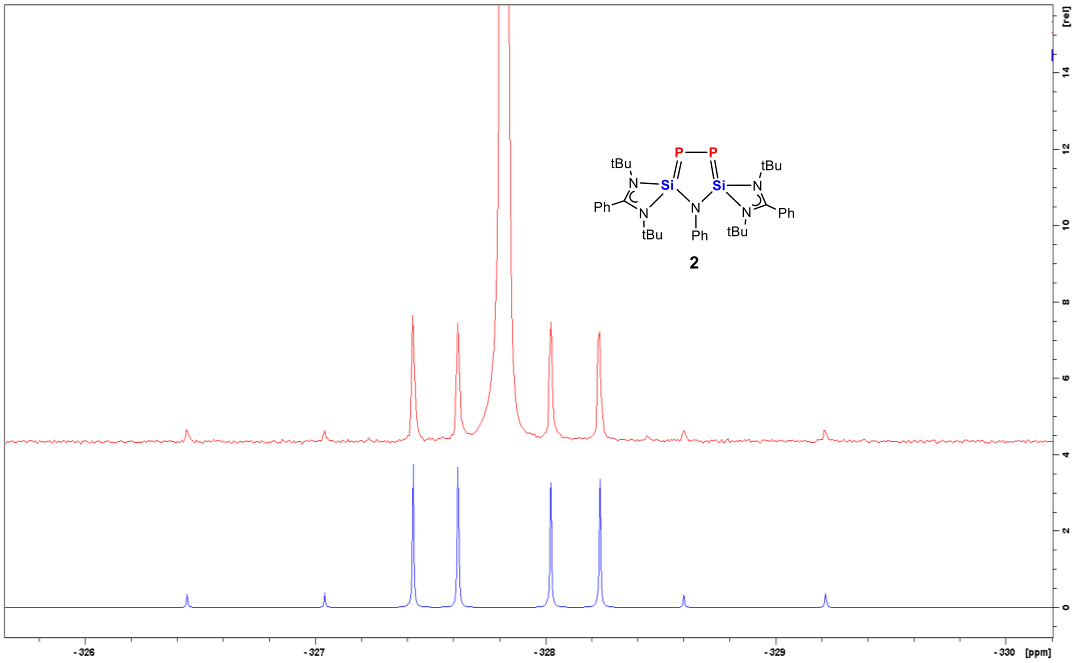

Figure S7. $^{31}P\{^1H\}$ NMR spectrum (161.98 MHz, THF-d_8/C_6D_6, 298 K) for **2** (top) and simulated spectrum (bottom, only the ^{29}Si isotopologue).

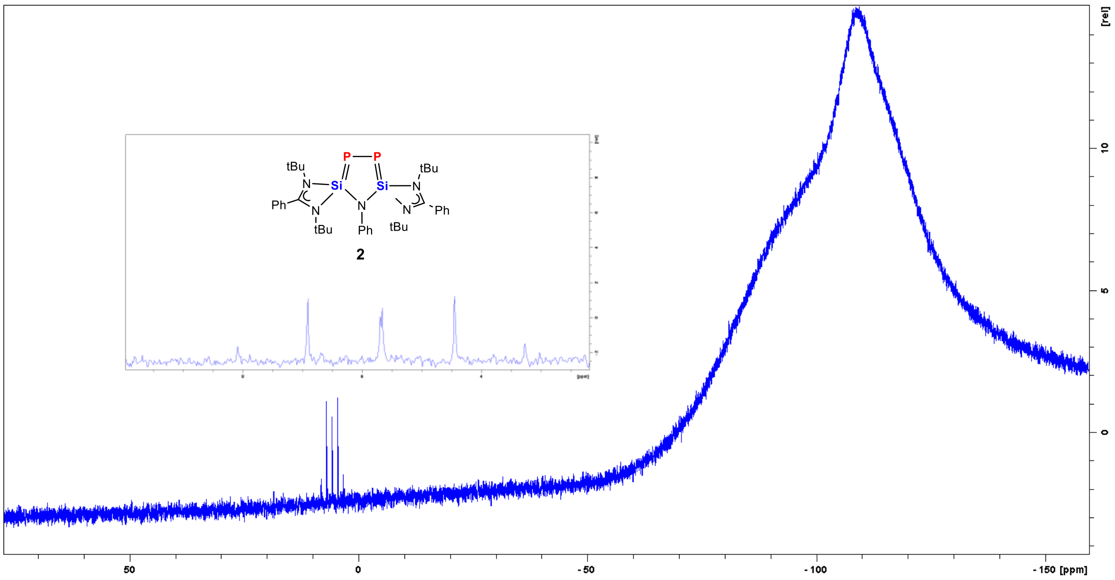

Figure S8. $^{29}Si\{^1H\}$ NMR spectrum (79.49 MHz, THF-d_8/C_6D_6, 298 K) of **2**.

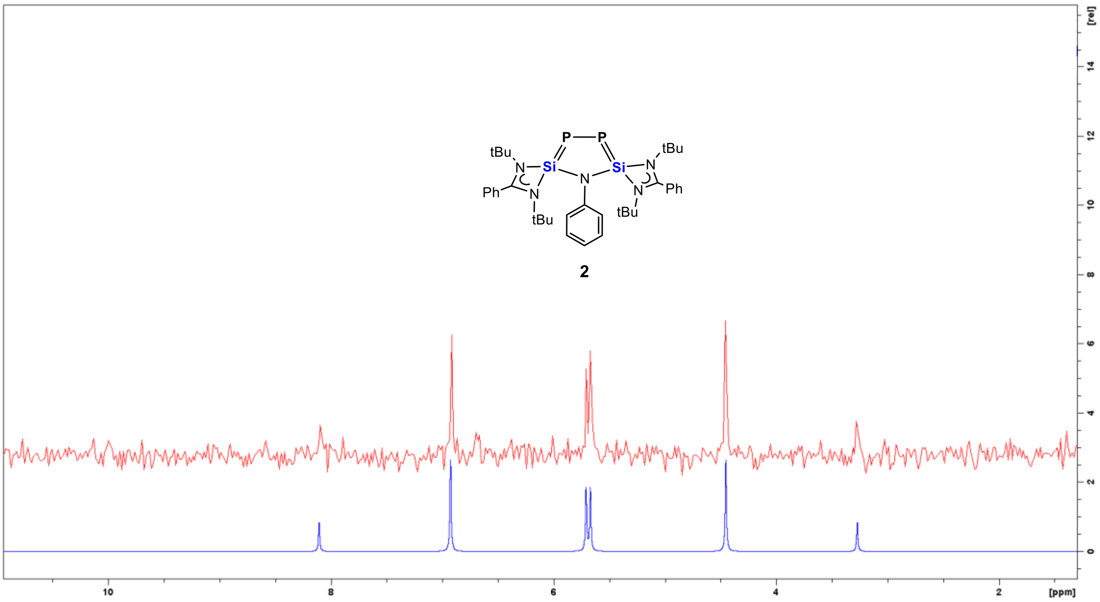

Figure S9. ^{29}Si{^{1}H} NMR spectrum (79.49 MHz, THF-d_8/C_6D_6, 298 K) of **2** (top) and simulated ^{29}Si NMR spectrum (bottom).

Simulated ^{31}P{^{1}H} and ^{29}Si{^{1}H} NMR spectra for 2 (^{29}Si isotopologue)

δ = -327.9628 (P1), -327.9463 (P2)

δ = 5.6871 (Si)

$^{1}J_{PP}$ = +/- 158.98736;

$^{1}J_{PSi}$ = 206.08895

$^{2}J_{PSi}$ = -9.52608

Spin simulation was done using Spinworks and NUMMRIT simulation. $^{1}J_{PSi}$ and $^{2}J_{PSi}$ have opposite signs. The sign of the $^{2}J_{PSi}$ coupling was set negative according to Berger, S.; Braun, S.; Kalinowski, H.-O. NMR Spectroscopy of the non-metallic elements, 1st ed.; Wiley, 1997.

Compound 3: 50 mL Et_2O was added to a mixture of **1** (0.91 g, 1.49 mmol) and P_4 (0.14 g, 1.14 mmol) at room temperature. After the reaction mixture was stirred overnight, the volatiles were removed under vacuum and the residue was washed with n-hexane (25 mL), yielding 0.77 g (0.55 mmol, 74% yield) of **3** as orange crystals. The X-ray analysis qualified crystals were obtained from THF solution. M.p. 132 °C (decomp.); **^{1}H NMR** (500.25 MHz, THF-d_8/C_6D_6, 298 K): δ(ppm) = 0.87 (s, 18 H, CH_3), 0.92 (s, 18 H, CH_3), 1.50 (s, 18 H, CH_3), 1.65 (s, 18 H, CH_3), 7.17 (*t*, 2H, J = 7.3Hz), 7.22 (*d*, 2H, J = 7.3Hz), 7.73 (*d*, 4H, J = 7.7Hz), 7.29 – 7.49 (*m*, 20H), 8.27 (*d*, 2H, J = 7.3Hz); **^{13}C{^{1}H} NMR** (125.79 MHz, THF-d_8/C_6D_6, 298 K): δ(ppm) = 32.24, 33.10, 33.25, 35.06 (C(CH_3)$_3$), 55.43, 55.66, 55.75, 57.90 (*C*Me$_3$), 124.86, 127.69, 129.34 (presumably 3x), 129.39, 129.48, 129.78, 130.18, 130.56, 131.80, 131.96 (*Ph*), 133.33 (quaternary *Ph*), 134.95 (*Ph*), 138.59, 151.53 (quaternary *Ph*), 167.01, 174.37 (N*C*N); **^{31}P{^{1}H} NMR** (81.01 MHz, THF-d_8/C_6D_6, 298 K): δ(ppm) = 40.2 (*ddm*, J = 192 Hz, 339 Hz, *P2(P5)*), -34.2 (*dm*, J = 192 Hz, *P3(P4)*), -247 (*dm*, J_{PP} = 15.6 Hz, 339 Hz, *P1(P6)*); **^{29}Si{^{1}H} NMR** (99.39 MHz, THF-d_8/C_6D_6, 298 K): δ(ppm) = 7.2 (*dd*, Si1, $^{1}J_{Si1P1}$ = 200Hz, $^{3}J_{Si1P2}$ = 10.5 Hz), -57.5 ppm (*m*, Si2); Elemental analysis calcd (%) for $C_{72}H_{102}N_{10}Si_4P_6$: (1405.85 g/mol) C 61.51, H 7.31, N 9.96, found: C 61.09, H 7.40, N 10.09. **IR** (cm^{-1}): 2966(*m*), 1590(*m*), 1546(*m*), 1480(*s*), 1445(*w*), 1404(*s*), 1393 (*s*), 1362(*s*), 1274(*w*), 1208(*vs*), 1114(*m*), 1091(*m*), 1071(*m*), 1022(*m*), 1003(*w*), 928(*vs*), 895(*vs*), 833(*vs*), 868(*vs*), 794(*m*), 765(*vs*), 733 (*w*), 722(*w*), 708(*vs*), 699(*vs*), 669(*vs*), 640(*s*), 615(*w*), 594(*s*). UV-Vis: 370nm, ε = 2.4x10^{6}.

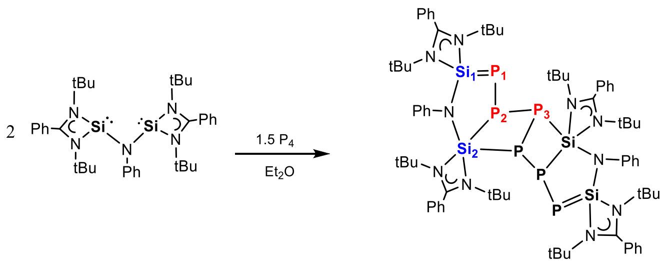

1 **3** (74%)

Scheme S3. Syntheses of **3**

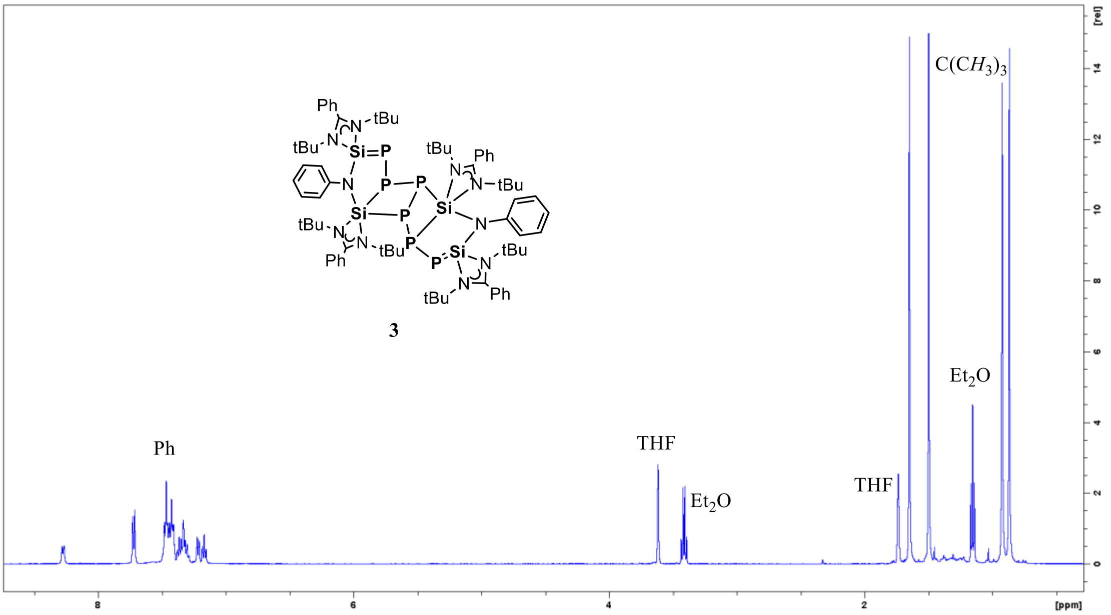

Figure S10. ^{1}H NMR spectrum (500.25 MHz, THF-d_8/C_6D_6, 298 K) of **3**.

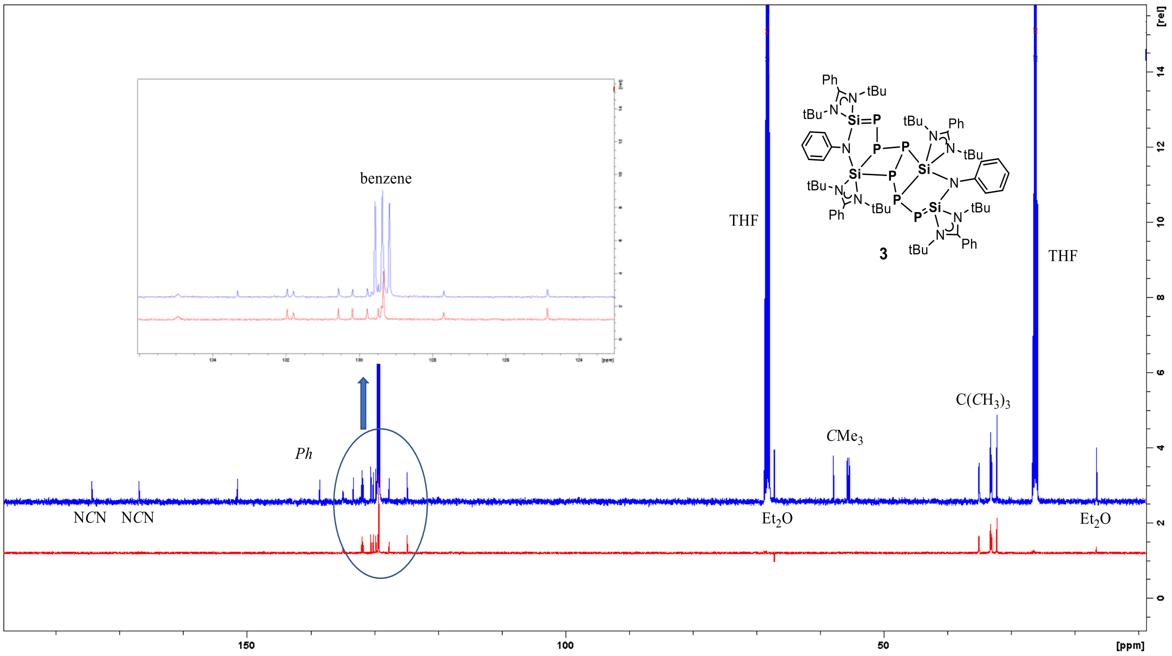

Figure S11. $^{13}C\{^{1}H\}$ (top) and DEPT-135 (bottom) NMR Spectra of **3** (100.61 MHz, THF-d_8/C_6D_6, 298 K).

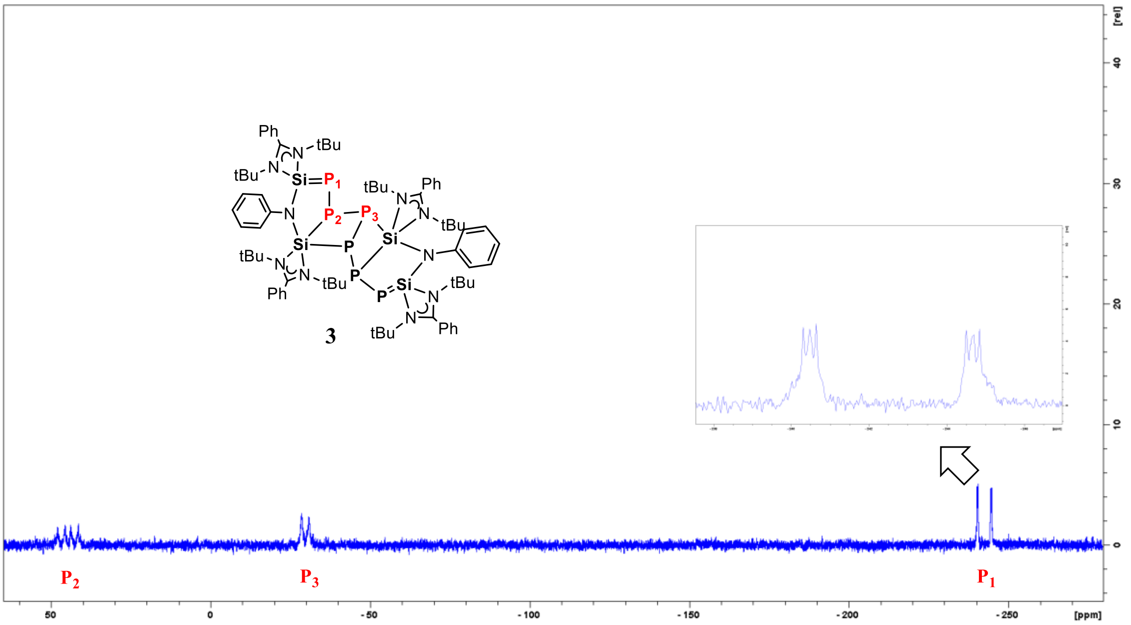

Figure S12. $^{31}P\{^{1}H\}$ NMR spectrum (81.01 MHz, THF-d_8/C_6D_6, 298 K) of **3**.

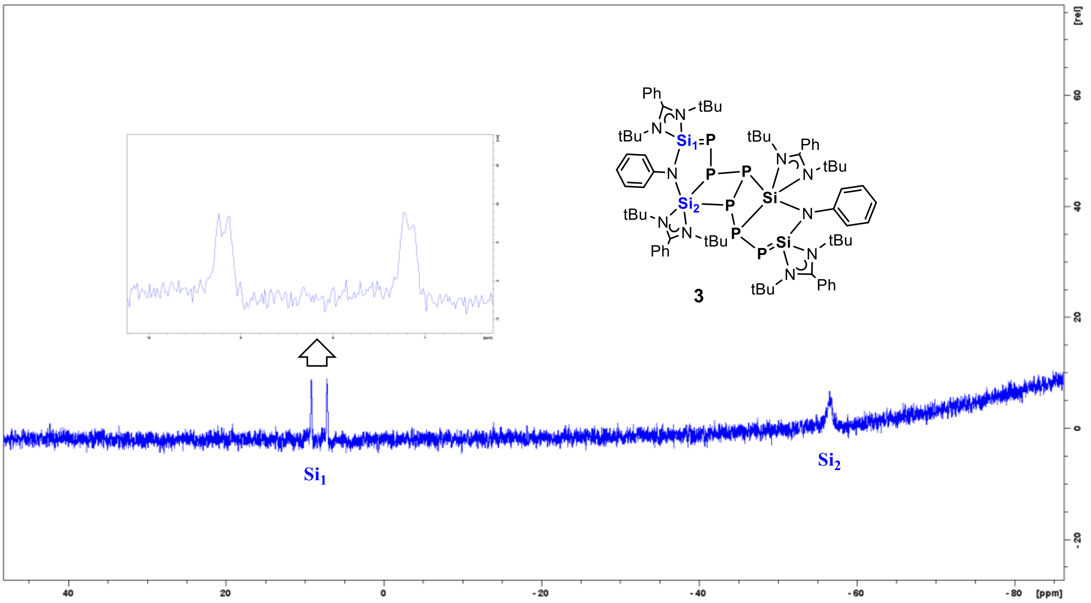

Figure S13. $^{29}Si\{^{1}H\}$ NMR spectrum (99.39 MHz, THF-d_8/C_6D_6, 298 K) of **3**.

Compound 4: A 20 mL THF solution of **1** (1.13 g, 1.85 mmol) was added to a 20 mL THF solution of P_4 (0.057 g, 0.46 mmol) at room temperature. The reaction mixture was stirred for 16 h. From the concentrated THF solution yellow crystals of **4** was obtained with yield of 85% (1.01 g, 0.79 mmol). M.p. 167 ^{o}C (decomp.); ^{1}H NMR (200.13 MHz, THF-d_8, 298 K): δ (ppm) 1.13 (*s*, 18 H, C*H*3), 1.28 (*s*, 18 H, C*H*3), 1.35 (*s*, 18 H, C*H*3), 1.51 (*s*, 18 H, C*H*3), 6.27 (*t*, 1H, *J* = 6.8Hz), 6.82 (*d*, 2H, *J* = 8.2 Hz), 6.93 (*t*, 2H, *J* = 7.7Hz), 7.09 – 7.65 (*m*, 23H), 8.46 (*d*, 1H, *J* = 8.0Hz), 9.29 (*t*, 1H, *J* = 7.8Hz); **^{13}C{^{1}H} NMR** (100.61 MHz, THF-d_8, 298 K): δ (ppm) = 32.59, 32.80,

32.88, 33.04 (C(*C*H_3)$_3$), 54.47, 54.92, 55.99, 56.49 (*C*Me_3), 112.89, 124.19, 125.30, 127.47, 127.96, 128.83, 128.85, 129.11, 129.15, 129.28, 129.33, 129.55, 129.70, 129.82, 129.91, 130.07, 130.61, 130.86, 131.00, 131.04, 131.11, 131.27, 131.43, 131.49, 132.14, 132.20 (*Ph*), 132.70, 134.56, 134.67, 136.14, 149.72, 162.21, 162.93, 172.63, 172.91, 176.39 (quaternary *Ph*) and (NCN); **^{31}P{^{1}H} NMR** (81.01 MHz, THF-*d*$_8$, 298 K): δ(ppm) = -263.8 (*d*, $^2J_{PP}$ = 47.6 Hz *P1*), -287.6 (*d*, $^2J_{PP}$ = 47.6 Hz *P2*); **^{29}Si{^{1}H} NMR** (79.49 MHz, THF-*d*$_8$, 298 K): δ (ppm) = 46.2 (*dd*, $^1J_{SiP}$ = 115Hz, $^1J_{SiP}$ = 188Hz *Si3*), 9.91 (*dd*, $^1J_{SiP}$ = 138Hz, $^3J_{SiP}$ = 7.8Hz *Si2*), -27.4 (*dd*, $^1J_{SiP}$ = 113Hz, $^3J_{SiP}$ = 10.9Hz *Si1*), -31.6 (*dd*, $^1J_{SiP}$ = 98.3Hz, $^3J_{SiP}$ = 3.5Hz *Si4*); Elemental analysis calcd (%) for $C_{72}H_{102}N_{10}Si_4P_2$: (1281.96 g/mol) C 67.46, H 8.02, N 10.93, found: C 66.98, H 8.23, N 11.24. **IR** (*cm*$^{-1}$): 2969(*m*), 1580(*m*), 1522(*w*), 1475(*s*), 1444(*w*), 1406(*vs*), 1361(*s*), 1304(*s*), 1275(*m*), 1210(*vs*), 1162(*w*), 1088(*w*), 1024(*m*), 1003(*m*), 927(*s*), 899(*m*), 842(*s*), 792(*s*), 766(*s*), 753(*m*), 744(*m*), 724(m), 708(*vs*), 700(*s*), 695(*s*), 662(*s*), 649(*w*), 640(*w*), 617(*m*), 612(*m*), 573(*s*). UV-Vis: 275 nm, ε = 5.9x10^5.

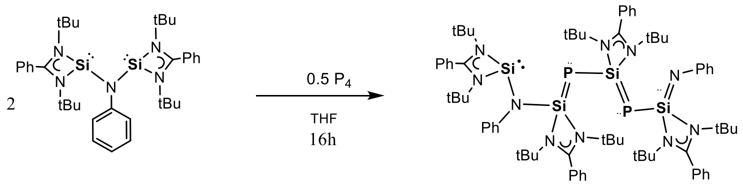

1 **4**

Scheme S4. Syntheses of **4**

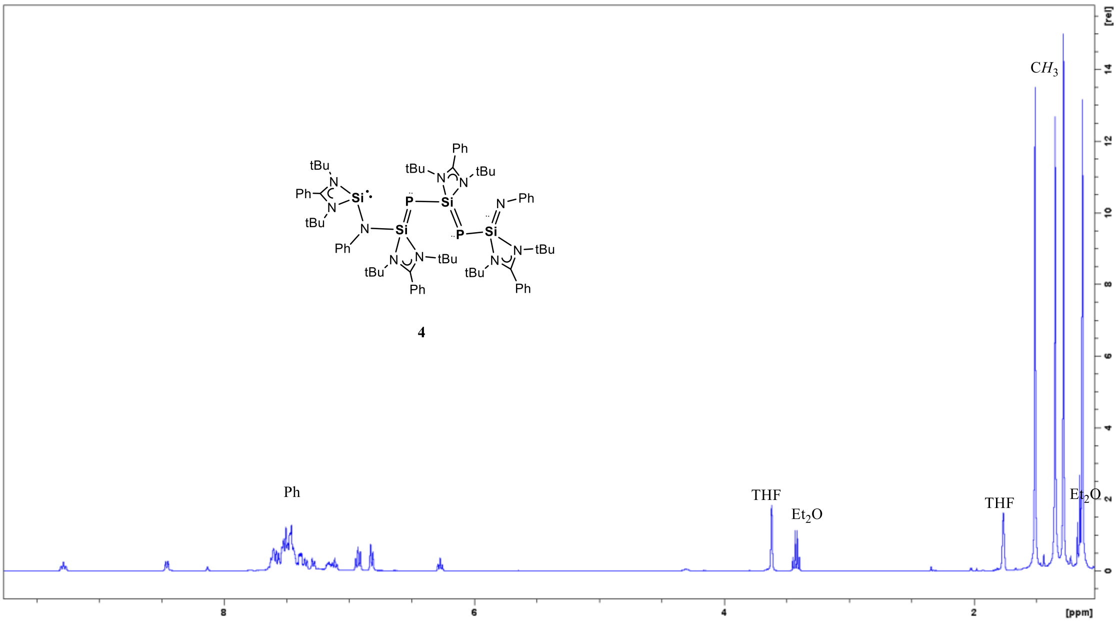

Figure S14. ^{1}H NMR spectrum (200.13 MHz, THF-*d*$_8$, 298 K) of **4**.

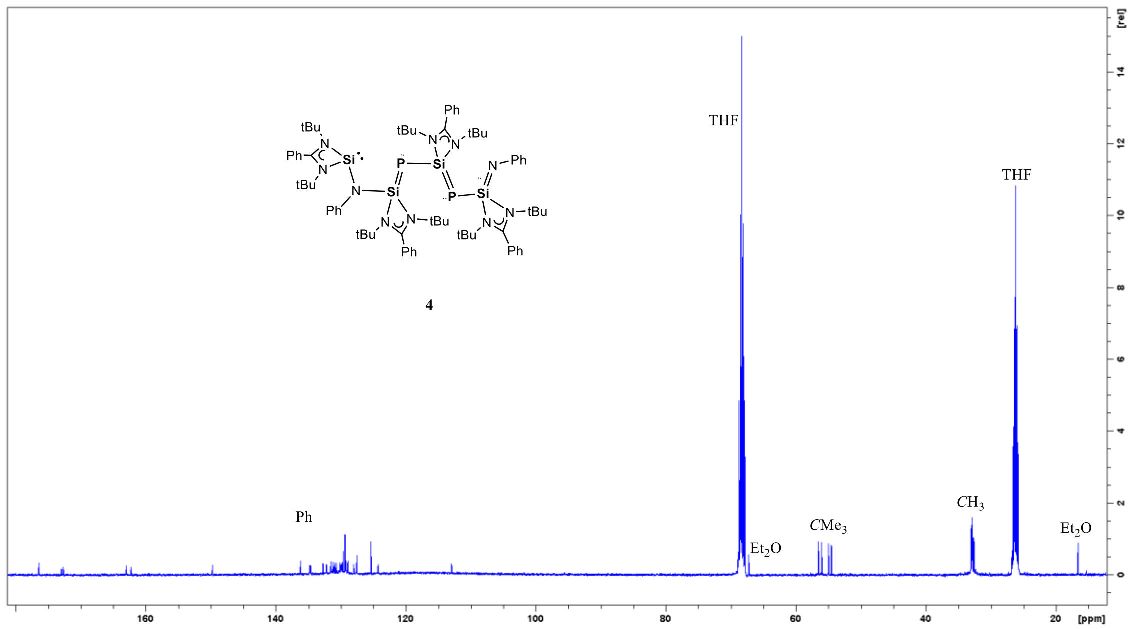

Figure S15. $^{13}C\{^1H\}$ NMR spectrum (100.61 MHz, THF-d_8, 298 K) of **4**.

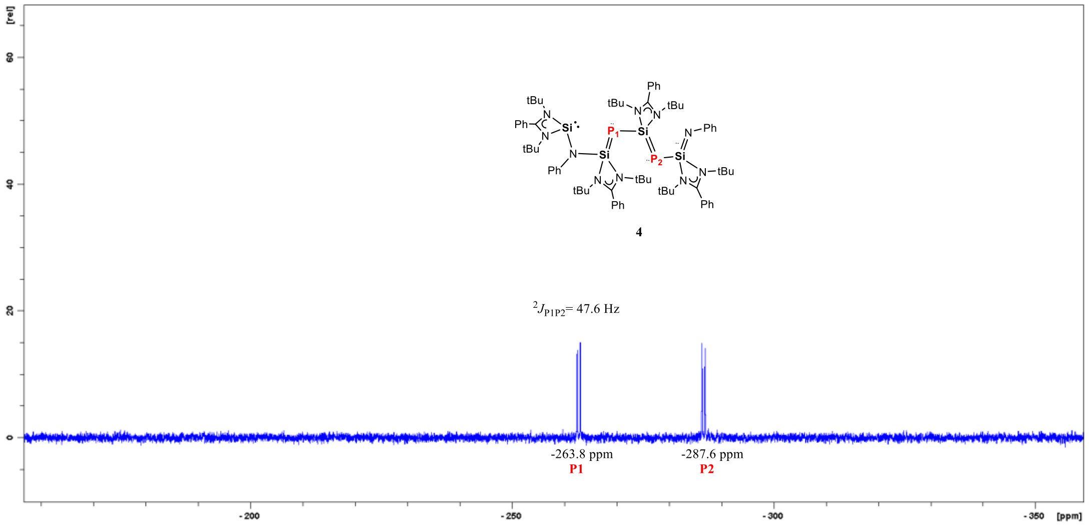

Figure S16. $^{31}P\{^1H\}$ NMR spectrum (81.01 MHz, THF-d_8, 298 K) of **4**.

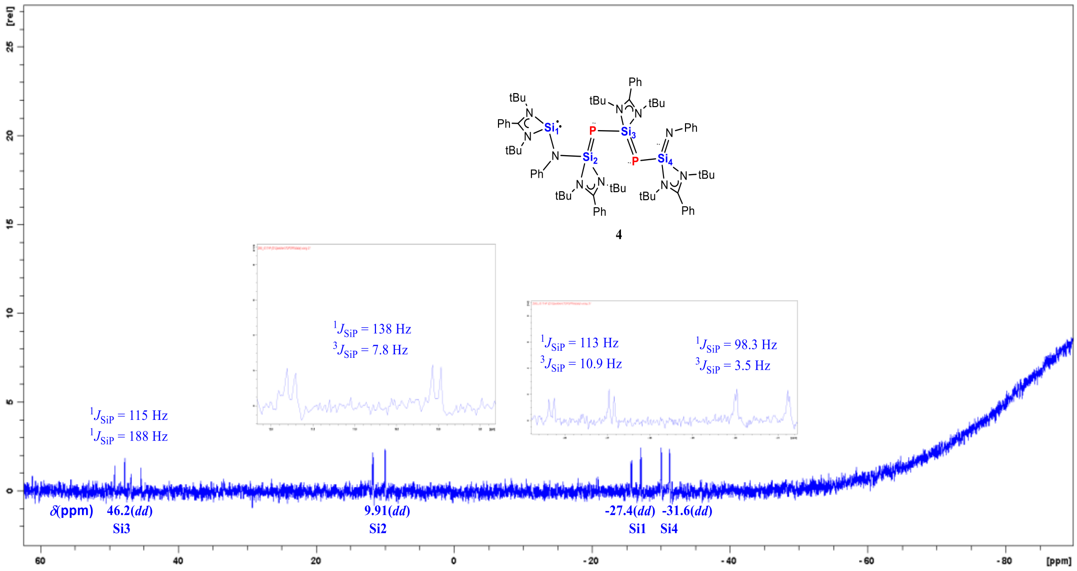

Figure S17. $^{29}Si\{^1H\}$ NMR spectrum (79.49 MHz, THF-d_8, 298 K) of **4**.

A3.2 Reactivity of 2, 3, and 4

Reactivity of 2 toward P_4 and 1 respectively:

a) The isolated compopund **2** reacts with P_4 to afford **3**: To a colorless solution of P_4 (0.0008 g, 0.0065 mmol) in 0.30 mL d_8-THF in an NMR tube was added a black solution of **2** (0.0175 g, 0.0260 mmol) in 0.20 ml d_8-THF at room temperature (molar ratio of **2** : P_4 = 4 : 1). After 1 h the reaction was completed and **3** was afforded quantitatively.

b) The isolated compound **2** reacts with **1** to afford **4**: when a yellow solution of **1** (0.0145 g, 0.0238 mmol) in 0.30 mL d_8-THF was added to a black solution of the isolated **2** (0.0160 g, 0.0238 mmol) in 0.30 ml d_8-THF in an NMR tube at room temperature. The reaction solution turned to yellow immediately. The $^{31}P\{^1H\}$ NMR spectrum of the reaction mixture confirmed the formation of **4**.

Reactivity of 3 toward bis(silylenes) 1:

Reaction of **3** with **1** to give **2**, which reacts with **1** further to give **4**: 0.50 mL d_8-THF was added to a mixture of **3** (0.0250 g, 0.0178 mmol) and **1** (0.0134 g, 0.0220 mmol) in an NMR tube at room temperature (molar ratio of **3** and **1** = 1.00 : 1.24). Since **3** needs four molar equivalents of **1** to convert to **4** completely (Figure S18), the added 1.24 molar equivalents of **1** can result only a mixture of unreacted **3**, reaction intermediate **2** and product **4**. As expected, the $^{31}P\{^1H\}$ NMR spectrum showed a signal at δ = -328.0 ppm for **2** along with the signals for product **4** and unreacted **3** as well as unidentified species (Figure S18). The subsequent addition of **1** (0.0490 g) to the mixture yielded complete conversion of **3** and **2** to **4**.

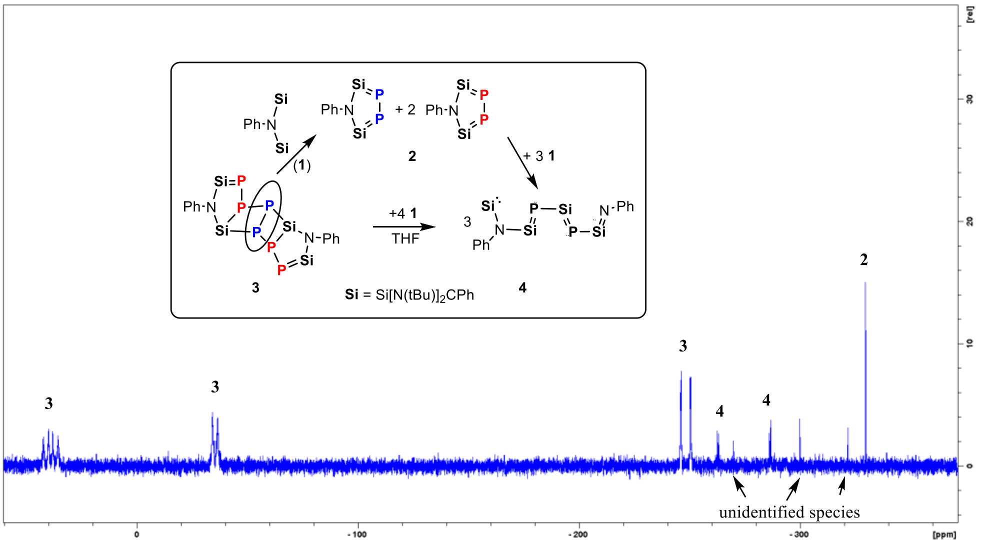

Figure S18. Compound **2** as intermediate in the conversion of **3** to **4** [**Si:** = Si(N(tBu))$_2$CPh] (**3** is in excess).

Reactivity of 3 toward (Xant)Si$_2$:

Reaction of **3** with (Xant)Si$_2$ to give **2** and **B**: 0.50 mL d_8-THF was added to a mixture of **3** (0.0032 g, 0.023 mmol) and (Xant)Si$_2$ (0.0066g, 0.0090 mmol) in an NMR tube at room temperature. After three days the $^{31}P\{^1H\}$ NMR spectrum of the resulting mixture exhibited a signal at δ = -282.4 ppm for **B** and a signal at δ = -328.0 ppm for **2** (Figure S19).

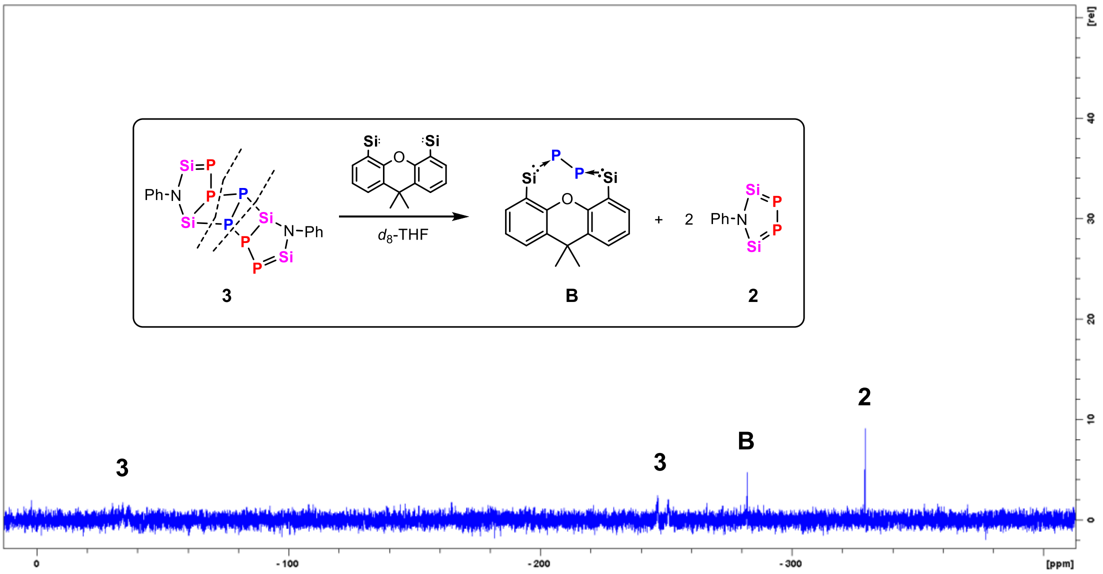

Figure S19. $^{31}P\{^1H\}$ spectrum of the reaction mixture of **3** and (Xant)**Si**$_2$ [**Si:** = Si(N(tBu))$_2$CPh].

Reactivity of 4 toward P_4:

*d*8-THF (0.50 mL) was added to an NMR tube with **4** (0.0110 g, 0.0085 mmol) and P_4 (0.0010 g, 0.0081 mmol) at room temperature. The $^{31}P\{^1H\}$ NMR spectrum of the reaction mixture showed that compound **4** converted to **3**.

A4. Details of the single crystal X-ray diffraction analyses

Table S1. Crystal data and structure refinement for **1**.

Empirical formula	C36 H51 N5 Si2	
Formula weight	609.99	
Temperature	150(2) K	
Wavelength	1.54184 Å	
Crystal system	Monoclinic	
Space group	$P2_1/c$	
Unit cell dimensions	a = 11.39940(10) Å	α= 90°.
	b = 17.8390(2) Å	β= 102.8530(10)°.
	c = 18.3255(3) Å	γ = 90°.
Volume	3633.19(8) $Å^3$	
Z	4	
Density (calculated)	1.115 Mg/m^3	
Absorption coefficient	1.109 mm^{-1}	
F(000)	1320	
Crystal size	0.320 x 0.210 x 0.120 mm^3	
Theta range for data collection	3.501 to 67.497°.	
Index ranges	-13<=h<=10, -21<=k<=21, -21<=l<=21	
Reflections collected	25325	
Independent reflections	6555 [R(int) = 0.0285]	
Completeness to theta = 67.497°	100.0 %	
Absorption correction	Semi-empirical from equivalents	
Max. and min. transmission	1.00000 and 0.33248	
Refinement method	Full-matrix least-squares on F^2	
Data / restraints / parameters	6555 / 0 / 400	
Goodness-of-fit on F^2	1.013	
Final R indices [I>2sigma(I)]	R1 = 0.0362, wR2 = 0.0892	
R indices (all data)	R1 = 0.0436, wR2 = 0.0952	
Extinction coefficient	n/a	
Largest diff. peak and hole	0.325 and -0.245 e.$Å^{-3}$	

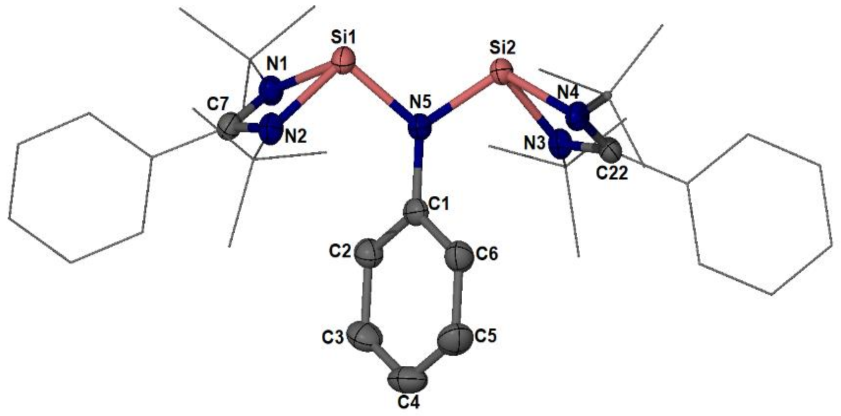

Figure S20. Molecular structure of **1**. Thermal ellipsoids are drawn at 50% probability level. Hydrogen atoms are omitted for clarity.

Table S2. Bond lengths [Å] and angles [°] for **1**.

Si2-N5	1.765(1)
Si2-N3	1.875(1)
Si2-N4	1.893(1)
Si1-N5	1.781(1)
Si1-N2	1.881(1)
Si1-N1	1.908(1)
N5-C1	1.425(2)
N3-C22	1.339(2)
N4-C22	1.334(2)
N1-C7	1.333(2)
N2-C7	1.336(2)
N5-Si2-N3	102.9(1)
N5-Si2-N4	106.2(1)
N3-Si2-N4	68.8(1)
N5-Si1-N2	101.6(1)
N5-Si1-N1	105.0(1)
N2-Si1-N1	68.6(1)
C1-N5-Si2	124.5(1)
C1-N5-Si1	126.0(1)
Si2-N5-Si1	109.4(1)
C22-N3-Si2	92.1(1)
C22-N4-Si2	91.4(1)
C7-N1-Si1	91.3(1)
C7-N2-Si1	92.4(1)

C2-C1-C6	117.5(1)
C2-C1-N5	121.6(1)
C6-C1-N5	120.9(1)
N4-C22-N3	105.7(1)
N1-C7-N2	106.4(1)

Table S3. Crystal data and structure refinement for 2.

Empirical formula	C36 H51 N5 P2 Si2	
Formula weight	671.93	
Temperature	150(2) K	
Wavelength	1.54184 Å	
Crystal system	Monoclinic	
Space group	$P2_1/c$	
Unit cell dimensions	a = 20.9035(5) Å	α= 90°.
	b = 15.2852(2) Å	β= 109.653(2)°.
	c = 18.3172(4) Å	γ = 90°.
Volume	5511.7(2) $Å^3$	
Z	4	
Density (calculated)	0.810 Mg/m^3	
Absorption coefficient	1.294 mm^{-1}	
F(000)	1440	
Crystal size	0.390 x 0.140 x 0.110 mm^3	
Theta range for data collection	3.661 to 67.499°.	
Index ranges	-22<=h<=25, -18<=k<=18, -21<=l<=16	
Reflections collected	39502	
Independent reflections	9922 [R(int) = 0.0395]	
Completeness to theta = 67.499°	99.9 %	
Absorption correction	Semi-empirical from equivalents	
Max. and min. transmission	1.00000 and 0.54814	
Refinement method	Full-matrix least-squares on F^2	
Data / restraints / parameters	9922 / 0 / 418	
Goodness-of-fit on F^2	1.078	
Final R indices [I>2sigma(I)]	R1 = 0.0429, wR2 = 0.1133	
R indices (all data)	R1 = 0.0520, wR2 = 0.1182	
Extinction coefficient	n/a	
Largest diff. peak and hole	0.372 and -0.337 e.$Å^{-3}$	

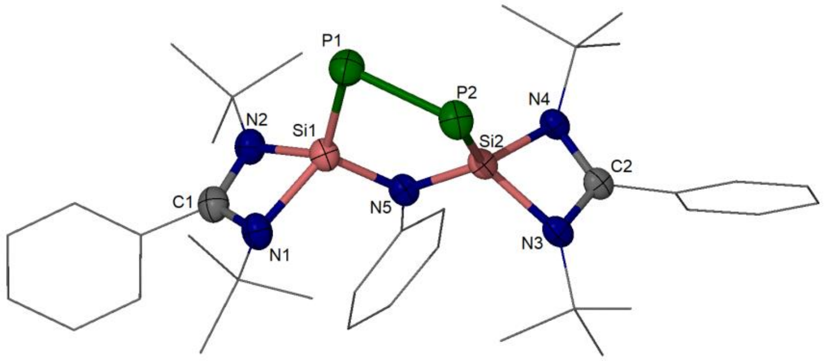

Figure S21. Molecular structure of **2**. Thermal ellipsoids are drawn at 50% probability level. Hydrogen atoms are omitted for clarity.

Table S4. Bond lengths [Å] and angles [°] for 2.

Si2-N5	1.736(1)
Si2-N3	1.829(1)
Si2-N4	1.853(1)
Si2-P2	2.115(1)
Si1-N5	1.749(1)
Si1-N2	1.820(1)
Si1-N1	1.855(1)
Si1-P1	2.108(1)
P2-P1	2.265(1)
N3-C2	1.340(2)
N4-C2	1.338(2)
N2-C1	1.339(2)
N1-C1	1.341(2)
N5-Si2-N3	111.8(1)
N5-Si2-N4	111.6(1)
N3-Si2-N4	71.0(1)
N5-Si2-P2	112.1(1)
N3-Si2-P2	120.5(1)
N4-Si2-P2	123.8 (1)
N5-Si1-N2	109.1(1)
N5-Si1-N1	109.6(1)

N2-Si1-N1	71.4 (1)
N5-Si1-P1	113.8(1)
N2-Si1-P1	120.8(1)
N1-Si1-P1	124.9(1)
Si2-P2-P1	95.5(1)
Si1-P1-P2	92.4(1)
C2-N3-Si2	91.8(1)
Si2-N5-Si1	109.6(1)
C2-N4-Si2	90.8(1)
C1-N2-Si1	91.8(1)
C1-N1-Si1	90.2(1)

Symmetry transformations used to generate equivalent atoms:

Table S5. Crystal data and structure refinement for **3**.

Empirical formula	C72 H102 N10 P6 Si4	
Formula weight	1405.81	
Temperature	150(2) K	
Wavelength	1.54184 Å	
Crystal system	Triclinic	
Space group	P-1	
Unit cell dimensions	a = 16.6668(7) Å	α= 86.969(4)°.
	b = 24.3254(10) Å	β= 70.922(4)°.
	c = 25.1425(12) Å	γ = 82.502(3)°.
Volume	9550.6(8) $Å^3$	
Z	4	
Density (calculated)	0.978 Mg/m^3	
Absorption coefficient	1.819 mm^{-1}	
F(000)	3000	
Crystal size	0.380 x 0.120 x 0.090 mm^3	
Theta range for data collection	2.625 to 67.500°.	
Index ranges	-19<=h<=13, -29<=k<=29, -30<=l<=29	
Reflections collected	67899	
Independent reflections	34245 [R(int) = 0.0817]	
Completeness to theta = 67.500°	99.6 %	
Absorption correction	Semi-empirical from equivalents	
Max. and min. transmission	1.00000 and 0.16222	
Refinement method	Full-matrix least-squares on F^2	
Data / restraints / parameters	34245 / 128 / 1760	

Goodness-of-fit on F^2	0.925
Final R indices [I>2sigma(I)]	R1 = 0.0792, wR2 = 0.1902
R indices (all data)	R1 = 0.1309, wR2 = 0.2240
Extinction coefficient	n/a
Largest diff. peak and hole	0.768 and -0.444 e.Å^{-3}

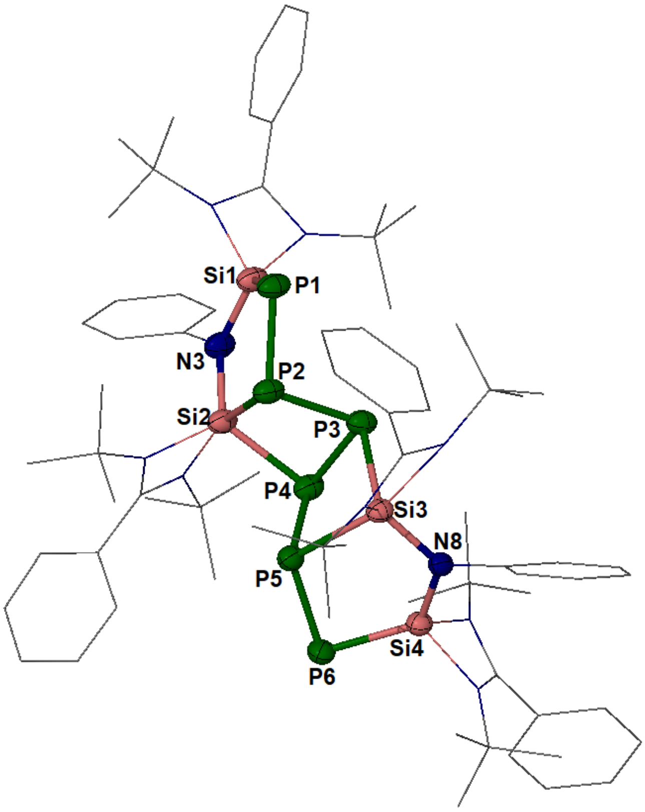

Figure S22. Molecular structure of for **3**. Thermal ellipsoids are drawn at 50% probability level. Hydrogen atoms are omitted for clarity.

Table S6. Bond lengths [Å] and angles [°] for **3**.

P5-P6	2.192(1)
P5-P4	2.249(2)
P5-Si3	2.330(2)
Si3-N8	1.770(3)
Si3-P3	2.275(1)
P6-Si4	2.121(2)
P4-P3	2.218(2)
P4-Si2	2.273(2)
P3-P2	2.234(2)
P2-P1	2.192(1)
P2-Si2	2.335(2)

P1-Si1	2.119(2)
Si1-N3	1.732(4)
Si1-N2	1.833(4)
Si1-N1	1.866(5)
Si2-N3	1.771(4)
Si2-N4	1.798(4)
Si2-N5	2.200(4)
P6-P5-P4	105.5(1)
P6-P5-Si3	107.7(1)
P4-P5-Si3	85.0(1)
N8-Si3-P3	116.8(1)
N8-Si3-P5	99.5(1)
P3-Si3-P5	87.8(1)
Si4-P6-P5	90.7(1)
P3-P4-P5	91.2(1)
P3-P4-Si2	87.2(1)
P5-P4-Si2	100.1(1)
P4-P3-P2	90.4(1)
P4-P3-Si3	87.0(1)
P2-P3-Si3	98.3(1)
P1-P2-P3	105.2(1)
P1-P2-Si2	108.2(1)
P3-P2-Si2	85.3(1)
Si1-P1-P2	91.6(1)
N3-Si1-N2	110.6(2)
N3-Si1-N1	110.2(2)
N3-Si1-P1	119.4(1)
N3-Si2-P4	120.8(1)
N3-Si2-P2	99.2(1)
N4-Si2-P2	109.3(2)
P4-Si2-P2	86.5(1)
Si4-N8-Si3	117.8(2)
Si1-N3-Si2	119.0(2)

Table S7. Crystal data and structure refinement for **4**.

Empirical formula	C72 H102 N10 P2 Si4	
Formula weight	1281.93	
Temperature	150(2) K	
Wavelength	1.54184 Å	
Crystal system	Triclinic	
Space group	P-1	
Unit cell dimensions	a = 15.2767(4) Å	α= 91.142(3)°.
	b = 15.8862(5) Å	β= 105.873(3)°.
	c = 18.9658(6) Å	γ = 95.745(2)°.
Volume	4399.7(2) $Å^3$	
Z	2	
Density (calculated)	0.968 Mg/m^3	
Absorption coefficient	1.269 mm^{-1}	
F(000)	1380	
Crystal size	0.470 x 0.250 x 0.190 mm^3	
Theta range for data collection	2.425 to 67.499°.	
Index ranges	-18<=h<=16, -18<=k<=19, -22<=l<=22	
Reflections collected	32145	
Independent reflections	15815 [R(int) = 0.0402]	
Completeness to theta = 67.499°	99.9 %	
Absorption correction	Semi-empirical from equivalents	
Max. and min. transmission	1.00000 and 0.56658	
Refinement method	Full-matrix least-squares on F^2	
Data / restraints / parameters	15815 / 0 / 817	
Goodness-of-fit on F^2	1.032	
Final R indices [I>2sigma(I)]	R1 = 0.0525, wR2 = 0.1452	
R indices (all data)	R1 = 0.0618, wR2 = 0.1542	
Extinction coefficient	n/a	
Largest diff. peak and hole	0.590 and -0.353 e.$Å^{-3}$	

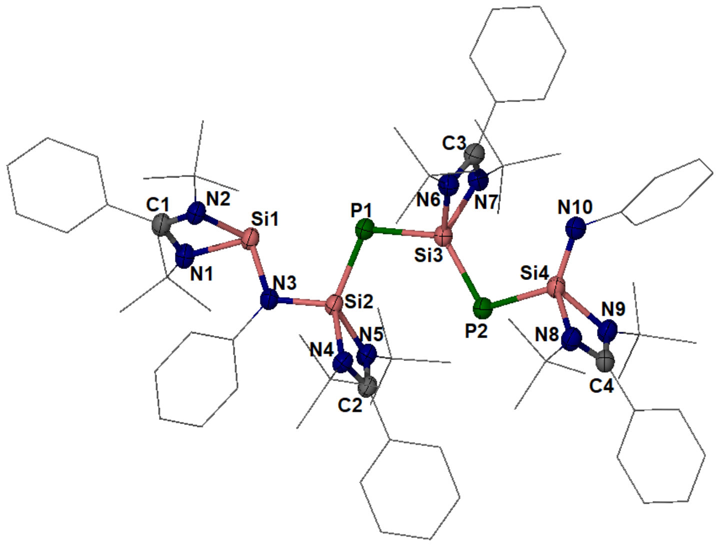

Figure S23. Molecular structure of for **4**. Thermal ellipsoids are drawn at 50% probability level. Hydrogen atoms are omitted for clarity.

Table S8. Bond lengths [Å] and angles [°] for **4**.

P2-Si3	2.145(1)
P2-Si4	2.178(1)
Si2-N3	1.728(2)
Si2-N4	1.832(1)
Si2-N5	1.846(1)
Si2-P1	2.127(1)
Si2-C2	2.284(1)
Si3-N7	1.845(2)
Si3-N6	1.850(2)
Si3-P1	2.186(1)
Si3-C3	2.299(2)
Si4-N10	1.625(2)
Si4-N9	1.871(2)
Si4-N8	1.881(2)
Si4-C4	2.326(2)
Si1-N3	1.803(1)
Si1-N1	1.879(2)
Si1-N2	1.903(2)
Si1-C1	2.333(2)
N5-C2	1.329(3)
N9-C4	1.328(3)
N4-C2	1.341(2)
N2-C1	1.327(3)

N1-C1	1.340(3)
N6-C3	1.336(3)
N8-C4	1.330(3)
N7-C3	1.322(3)
Si3-P2-Si4	104.1(1)
N3-Si2-N4	108.3(1)
N3-Si2-N5	112.1(1)
N4-Si2-N5	71.5(1)
N3-Si2-P1	108.6(1)
N4-Si2-P1	128.6(1)
N5-Si2-P1	123.1(1)
N3-Si2-C2	116.7(1)
N4-Si2-C2	35.9(1)
N5-Si2-C2	35.6(1)
P1-Si2-C2	134.7(1)
N7-Si3-N6	70.6(1)
N7-Si3-P2	119.7(1)
N6-Si3-P2	120.1(1)
N7-Si3-P1	104.4(1)
N6-Si3-P1	105.5(1)
P2-Si3-P1	123.9(1)
N7-Si3-C3	35.1(1)
N6-Si3-C3	35.5(1)
P2-Si3-C3	127.4(1)
P1-Si3-C3	108.8(1)
Si2-P1-Si3	108.7(1)
N10-Si4-N9	114.2(1)
N10-Si4-N8	118.6(1)
N9-Si4-N8	69.6(1)
N10-Si4-P2	125.8(1)
N9-Si4-P2	108.1(1)
N8-Si4-P2	106.7(1)
N10-Si4-C4	124.2(1)
N9-Si4-C4	34.8(1)
N8-Si4-C4	34.9(1)
P2-Si4-C4	109.8(1)
N3-Si1-N1	101.3(1)
N3-Si1-N2	105.0(1)

N1-Si1-N2	68.8(1)
N3-Si1-C1	111.8(1)
N1-Si1-C1	35.0(1)
N2-Si1-C1	34.7(1)
C2-N5-Si2	90.5(1)
C4-N9-Si4	91.7(1)
C2-N4-Si2	90.8(1)
C1-N2-Si1	90.7(1)
Si2-N3-Si1	115.7(1)
C1-N1-Si1	91.4(1)
C3-N6-Si3	90.9(1)
C4-N8-Si4	91.2(1)
C3-N7-Si3	91.6(1)
N5-C2-N4	107.1(1)
N5-C2-Si2	53.9(1)
N4-C2-Si2	53.3(1)
N9-C4-N8	107.3(2)
N9-C4-Si4	53.5(1)
N8-C4-Si4	53.9(1)
N2-C1-N1	106.6(2)
N2-C1-Si1	54.6(1)
N1-C1-Si1	53.6(1)
N7-C3-N6	106.9(2)
N7-C3-Si3	53.4(1)
N6-C3-Si3	53.6(1)

B Computational Section

Computational details. All the calculations were performed using the Gaussian 16 software package.[4] Geometry optimization of the compounds was conducted at the TPSS-D3BJ [5] density functional theory level, according to the best agreement with the metric data from X-ray structure analyses (Table S9, S10, S11). The Def2-SVP [6] basis set is used to describe C, N, H atoms, whereas ma-TZVP [7-8] basis set is used to Si and P atoms. In addition, frequency calculations are carried out at the same level of theory to confirm the stationary points are minima with no imaginary frequencies. Furthermore, the B97-2[9]/Def2-TZVP[10] method is used to calculate the ^{31}P NMR chemical shifts, where the solvent effect (solvent = THF) is taken into account by SMD model. The calculated ^{31}P absolute shielding constants are converted to ^{31}P NMR chemical shifts, with 85% water solution of H_3PO_4 as reference. Here, we use $\sigma(H_3PO_4)$ = 328.35 ppm suggested by Jameson *et al.*[11] The calculated ^{29}Si absolute shielding constants are converted to ^{29}Si NMR chemical shifts, with that of tetramethylsilane (TMS) calculated at the same level (σ(TMS) = 338.2 ppm) as reference. Viewing of optimized structures and rendering of molecular orbitals were performed using the program CYLview[12] and VMD,[13] respectively. NMR spectra were drawn in the Multiwfn program.[14] EDDB and NICS calculations were performed at the CAM-B3LYP/def2-TZVP level. Here, the more representative NICS(1)$_{ZZ}$ was employed to aromaticity calculation because it has been proven as a good index for both the S_0 and T_1 states.[15] Since the 6/5MR in compounds **D** and **2** are nonplanar, their NICS(1)$_{ZZ}$ values are averaged at 1Å above and below the ring center. For TD-DFT, PBE0 is used for UV/vis absorption simulation calculation at gas phase because König *et al.*[16] indicates that PBE0 performs well in the calculation of excitation energy.

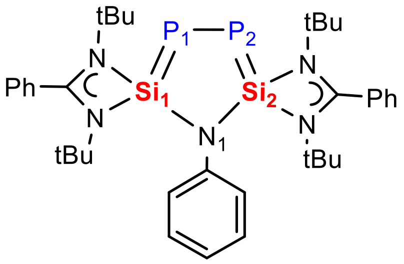

Table S9. Key distances (Å) of experimental and DFT-optimized structures of compound **2**.[a]

Functional	Exp.	TPSS	PBE0	B3LYP
N1-Si1	1.736	1.752	1.743	1.745
Si1-P1	2.114	2.113	2.103	2.106
P1-P2	2.265	2.281	2.271	2.297
P2-Si2	2.108	2.113	2.103	2.106
Si2-N	1.749	1.752	1.743	1.745
N1-C	1.432	1.408	1.398	1.407
RD(%)[a]	0	0.75	0.83	0.88

[a] RD = $\frac{\sum_{i=1}^{n} \frac{|BL(DFT) - BL(Exp)|}{BL(Exp)} * 100\%}{n}$, BL means bond length.

NMR analysis. According to the DFT calculation, the peak located at -340.1 ($\sigma_{exp.}$ = -330 ppm of **2**) is assigned to the P1 and P2 atoms (Figure S24).

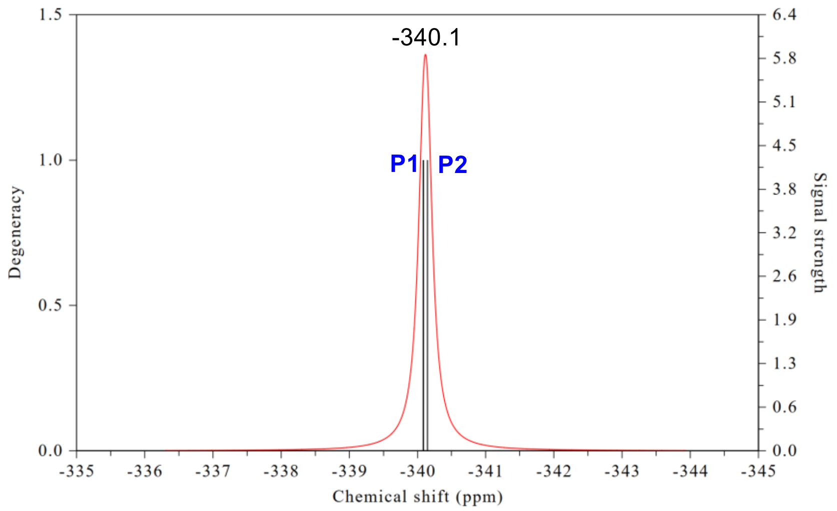

Figure S24. Calculated ^{31}P NMR spectrum of **2**.

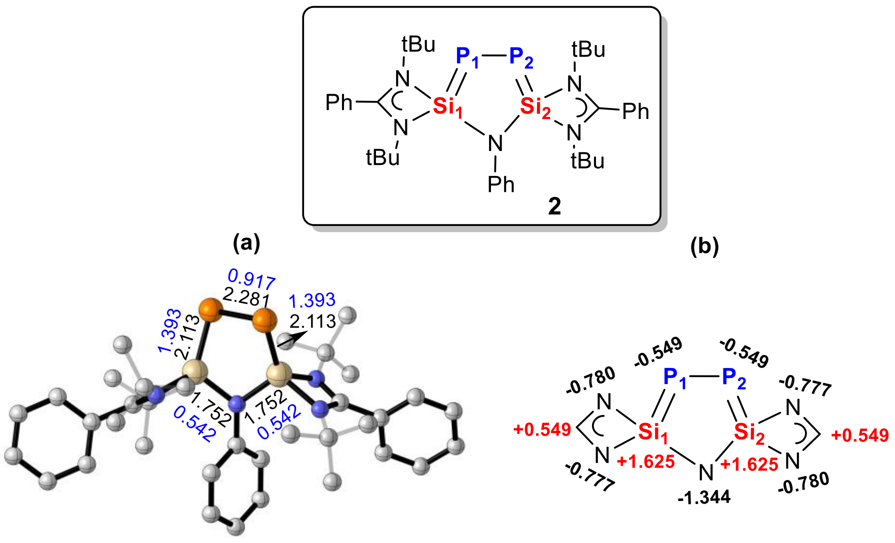

Figure S25. Structural parameters of compound **2** at TPSS-D3BJ / def2-SVP ~ ma-TZVP level. Bond length (a, black) are in Å; Wiberg bond index (a, blue). (b)The natural population analysis of the charges in **2**. Hydrogen atoms and substituents are omitted for clarity in the 3D structure and the NPA diagram, respectively.

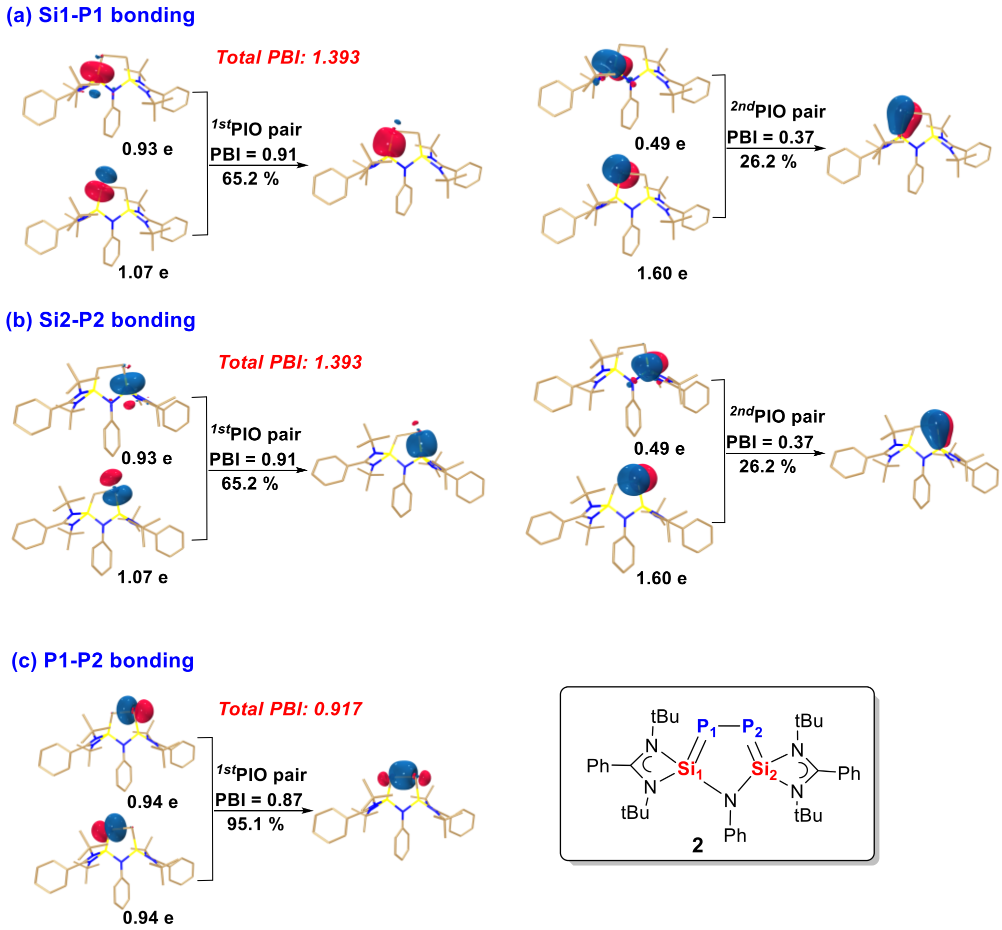

Figure S26. PIO analysis on the bonding modes of between Si/P and P atoms in compound **2**. Hydrogen atoms in 3D structures are omitted for clarity. The PIO analysis is performed by cutting the Si/P-P bonds. The isosurface 0.050 au is plotted.

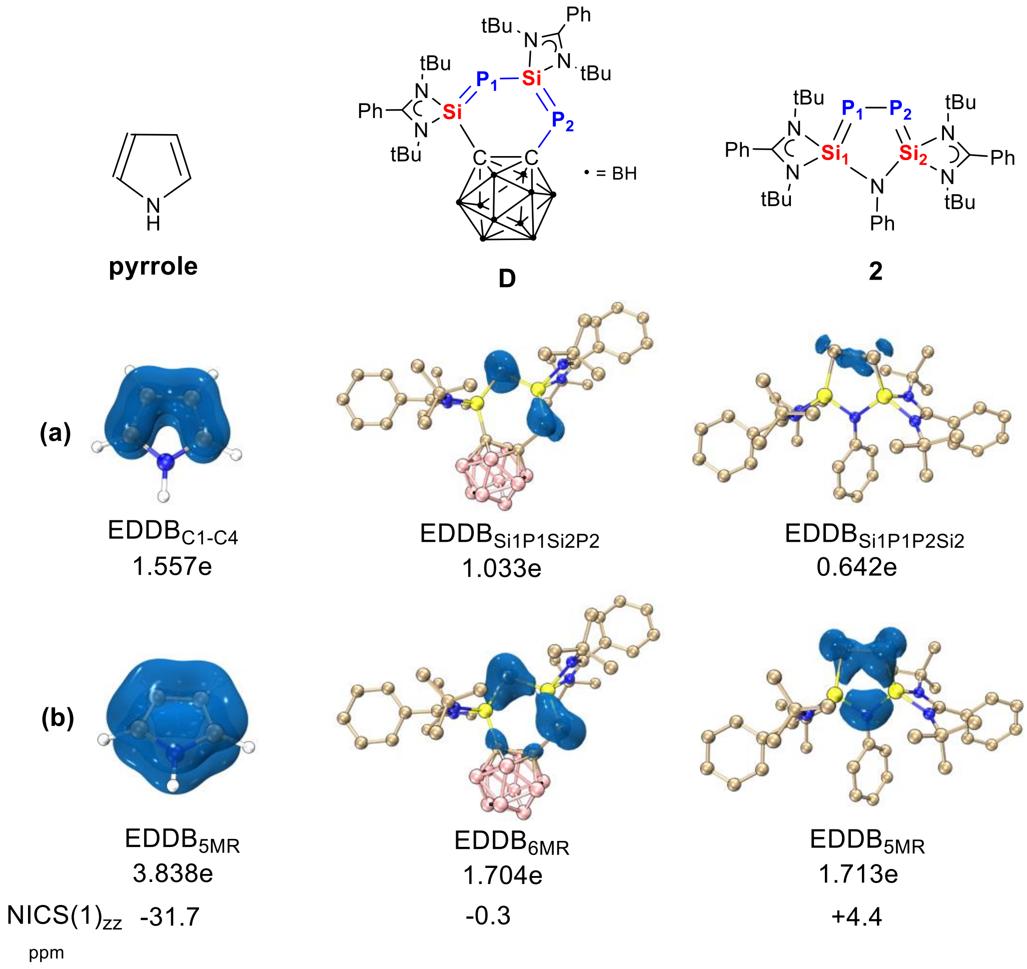

Figure S27. The electron density of delocalized bonds (EDDB) analyses for **pyrrole**, **D**, and **2**. Delocalized electrons of the specified fragments are listed below the corresponding plots. Hydrogen atoms in 3D structures are omitted for clarity (isovalue = 0.005 a.u.).

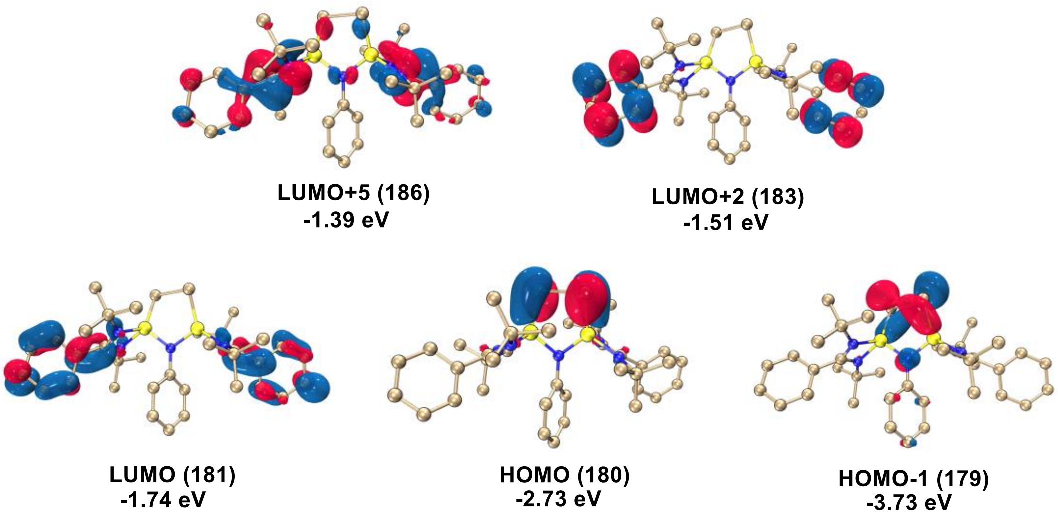

Figure S28. HOMO and LUMO orbitals of the compound **2**. Hydrogen atoms in 3D structures are omitted for clarity. The isosurface 0.030 a.u. is plotted.

The calculation of UV absorption spectrum. According to the DFT calculation, the maximum absorption wavelength located at 600.2 nm ($\lambda_{exp.}$ = 568 nm) of **2** (Figure S29), and the oscillator strength (f) is 0.0361. The HOMO was mainly localized on the five-membered ring moiety, whereas the LUMO was mostly localized on the benzene rings of the both side (Figure S28). The computed absorption band (λ = 600.2) can be assigned to the electronic transitions HOMO→LUMO+2 (19.2%), HOMO→LUMO+5 (72.2%). In addition, we also located an absorption peak at 421.2 nm ($\lambda_{exp.}$ = 426 nm) with f = 0.0151, and the computed absorption band can be assigned to the electronic transition HOMO-1→LUMO (77.8%).

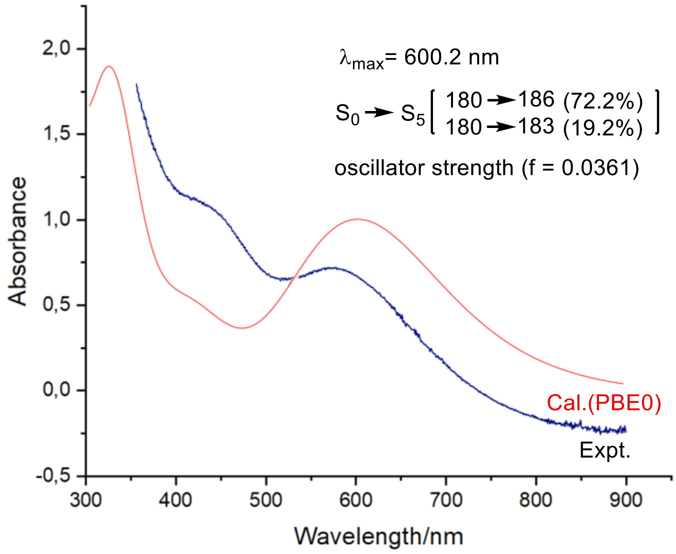

Figure S29. The UV/vis absorption spectrums of the compound **2**. The basis set is def2-TZVP.

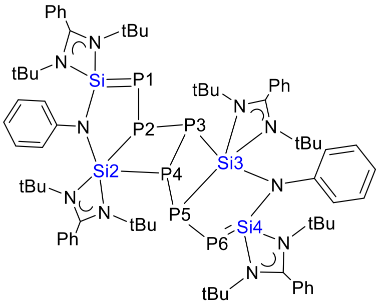

3

Table S10 Key distances (Å) of experimental and DFT-optimized structures of compound **3**.[a]

Functional	Exp.	PBE0	TPSS	B3LYP
Si1-P1	2.119	2.117	2.124	2.119
P1-P2	2.192	2.18	2.196	2.191
P2-Si2	2.335	2.344	2.349	2.333
P2-P3	2.234	2.222	2.239	2.247
P3-P4	2.218	2.19	2.205	2.234
P4-Si2	2.273	2.287	2.292	2.286
RD(%)[a]	0	0.4	0.3	0.6

[a] RD = $\frac{\sum_{i=1}^{n} \frac{|BL(DFT) - BL(Exp)|}{BL(Exp)} * 100\%}{n}$, BL means bond length.

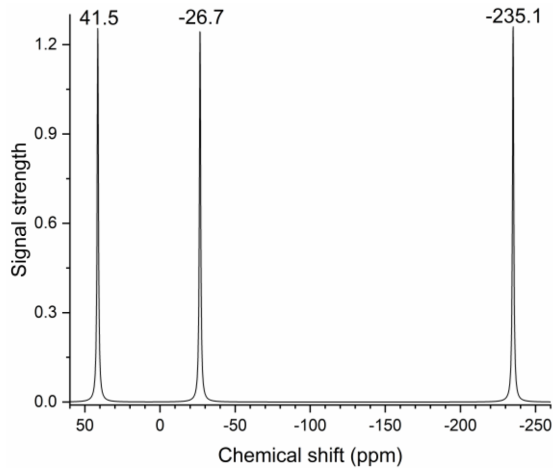

Figure S30. Calculated ^{31}P NMR Spectrum of **3**. According to the DFT calculation, the peaks located at 41.5 ($\delta_{exp.}$ = 41.2), -26.7 ($\delta_{exp.}$ = -33.2) and -235.1 ($\delta_{exp.}$ = -246) ppm of **3** are assigned to the P2 (P5), P3 (P4), and P1 (P6) atoms, respectively.

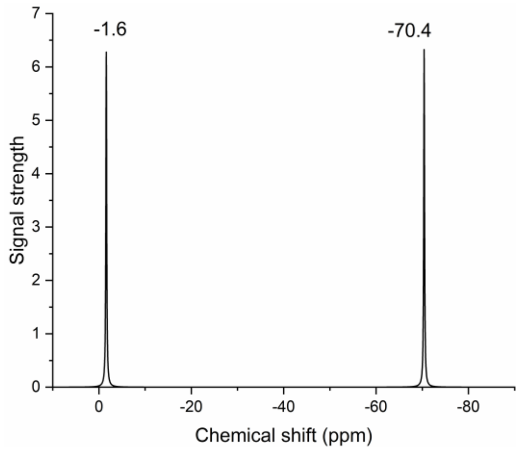

Figure S31. Calculated ^{29}Si NMR Spectrum of **3**. According to the DFT calculation, the ^{29}Si NMR peaks located at -1.6 and -70.4 ppm of **3** are assigned to the Si1 (Si4) and Si2 (Si3) atoms, respectively.

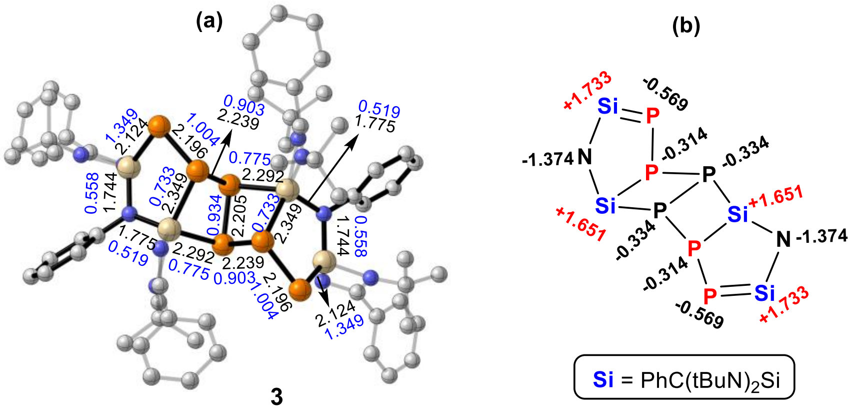

Figure S32. Structural parameters of compound **3** at TPSS-D3BJ / def2-SVP ~ ma-TZVP level. Bond length (a, black) are in Å; Wiberg bond index (a, blue). The natural population analysis of the charges in **3** (b). Hydrogen atoms and substituents are omitted for clarity in the 3D structure and the NPA diagram, respectively.

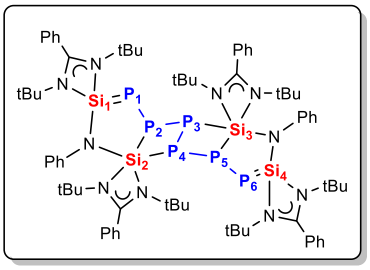

3

Si1-P1 bonding

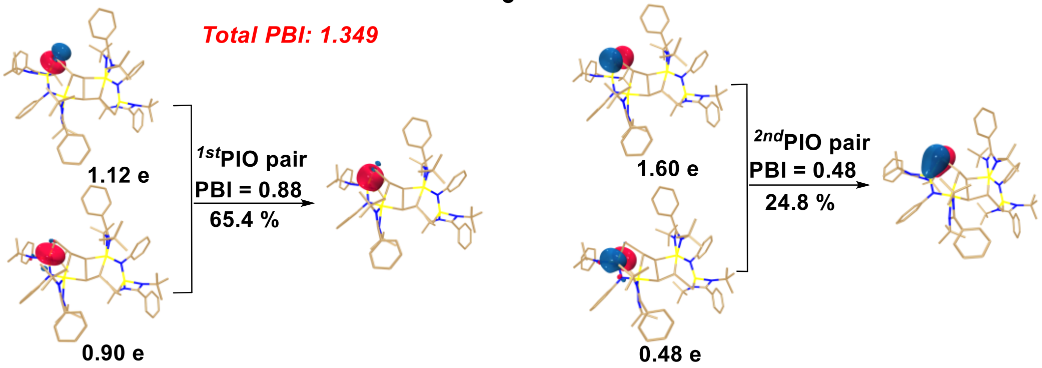

Figure S33 PIO analysis on the bonding modes of Si1-P1 in compound **3**. Hydrogen atoms in 3D structures are omitted for clarity. The PIO analysis is performed to the Si and P atoms. Each PIO pair leads to a bonding PIMO (principal interacting molecular orbital). The PBI quantifies the strength of the interaction. The isosurface 0.050 au is plotted.

Si2-P2 bonding

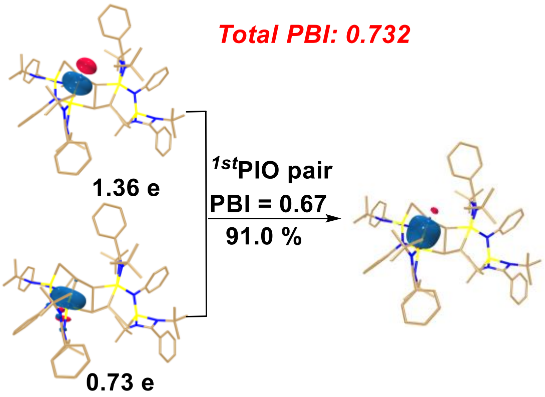

Figure S34. PIO analysis on the bonding modes of Si2-P2 in compound **3**. Hydrogen atoms in 3D structures are omitted for clarity. The PIO analysis is performed to the Si and P atoms. Each PIO pair leads to a bonding PIMO. The PBI quantifies the strength of the interaction. The isosurface 0.050 au is plotted.

Si2-P4 bonding

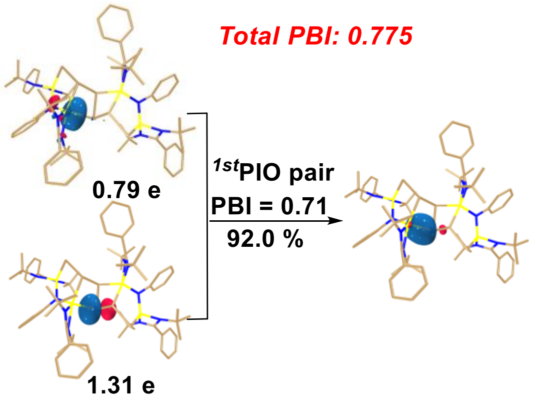

Figure S35. PIO analysis on the bonding modes of Si2-P4 in compound **3**. Hydrogen atoms in 3D structures are omitted for clarity. The PIO analysis is performed to the Si and P atoms. Each PIO pair leads to a bonding PIMO. The PBI quantifies the strength of the interaction. The isosurface 0.050 au is plotted.

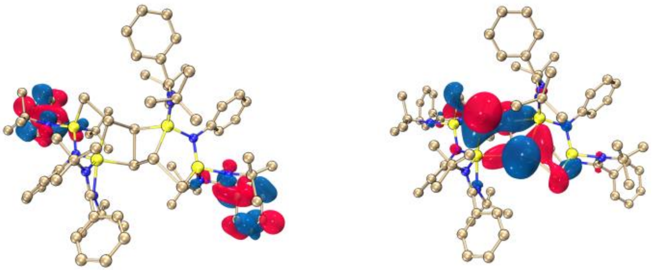

LUMO
-1.73 eV

HOMO
-3.24 eV

Figure S36. HOMO and LUMO orbitals of the compound **3**. Hydrogen atoms in 3D structures are omitted for clarity. The isosurface 0.050 au is plotted.

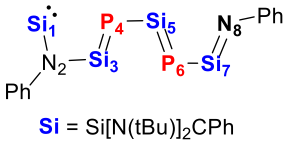

4

Table S11 Key distances (Å) of experimental and DFT-optimized structures of compound **4**.[a]

Functional	Exp.	TPSS
Si1-N2	1.803	1.810
N2-Si3	1.728	1.739
Si3-P4	2.127	2.124
P4-Si5	2.186	2.189
Si5-P6	2.145	2.144
P6-Si7	2.178	2.165
Si7-N8	1.625	1.644
RD(%)[a]	0	0.4

[a] RD = $\frac{\sum_{i=1}^{n}\frac{|BL(DFT)-BL(Exp)|}{BL(Exp)}*100\%}{n}$, BL means bond length.

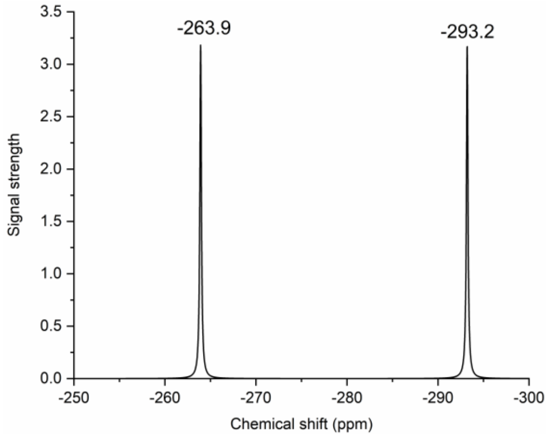

Figure S37. Calculated ^{31}P NMR Spectrum of **4**. According to the DFT calculation, the ^{31}P NMR peaks located at -263.9 ($\delta_{exp.}$ = -262.8), -293.2 ($\delta_{exp.}$ = -286.6) ppm of **4** are assigned to the P4 and P6 atoms, respectively.

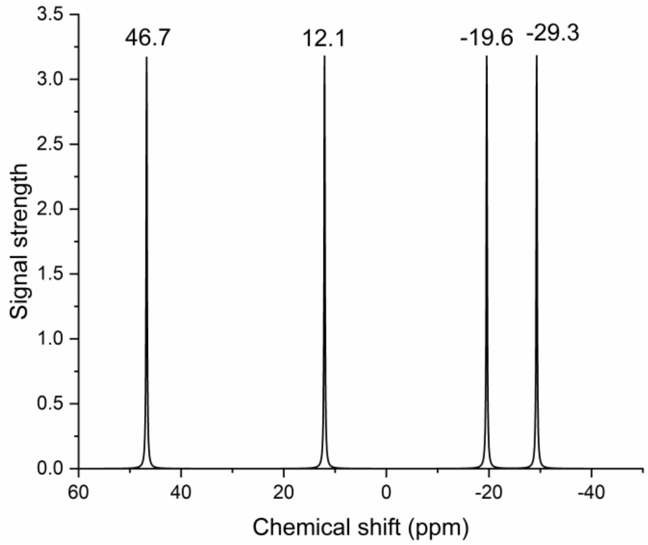

Figure S38. Calculated ^{29}Si NMR Spectrum of **4**. According to DFT calculations, the ^{29}Si NMR peaks located at 46.7 ($\delta_{exp.}$ = 47.2), 12.1 ($\delta_{exp.}$ = 10.9), -19.6 ($\delta_{exp.}$ = -26.4) and -29.3 ($\delta_{exp.}$ = -30.6) ppm of **4** are assigned to the Si5, Si3, Si7 and Si1 atoms, respectively.

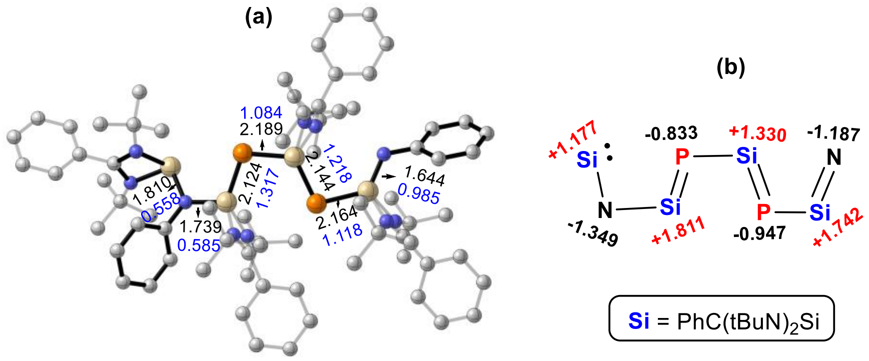

4

Figure S39. Structural parameters of compound **4** at TPSS-D3BJ / def2-SVP ~ ma-TZVP level. Bond length (a, black) are in Å; Wiberg bond index (a, blue). The natural population analysis of the charges in **4** (b). Hydrogen atoms and substituents are omitted for clarity in the 3D structure and the NPA diagram, respectively.

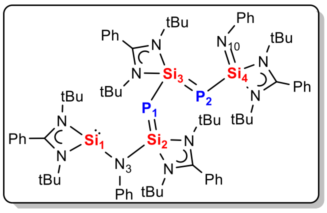

4

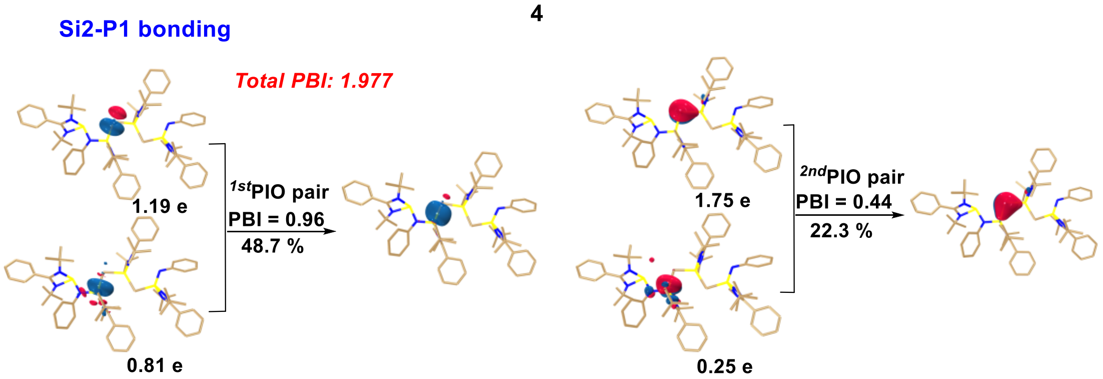

Figure S40. PIO analysis on the bonding modes of Si2-P1 bond in compound **4**. Hydrogen atoms in 3D structures are omitted for clarity. The PIO analysis is performed by cutting the Si2-P1 bond. Each PIO pair leads to a bonding PIMO. The PBI quantifies the strength of the interaction. The isosurfaces with the isovalue of 0.050 au are plotted.

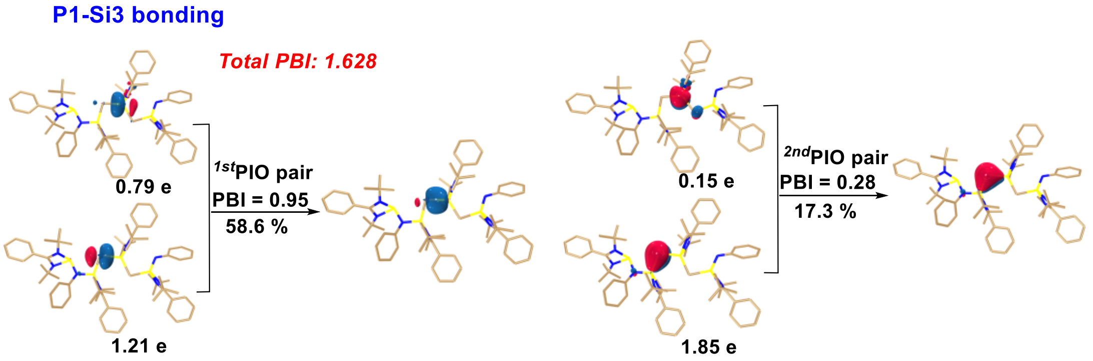

Figure S41. PIO analysis on the bonding modes of P1-Si3 bond in compound **4**. Hydrogen atoms in 3D structures are omitted for clarity. The PIO analysis is performed by cutting the P4-Si5 bond. Each PIO pair leads to a bonding PIMO. The PBI quantifies the strength of the interaction. The isosurfaces with the isovalue of 0.050 au are plotted.

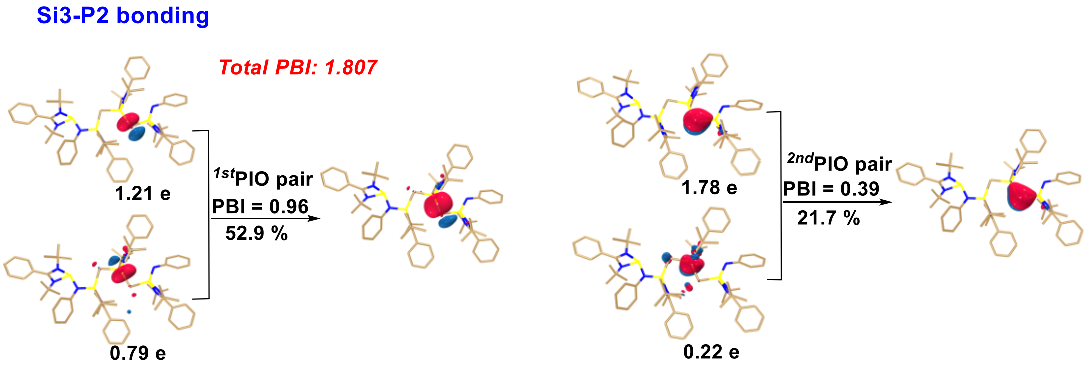

Figure S42. PIO analysis on the bonding modes of Si3-P2 bond in compound **4**. Hydrogen atoms in 3D structures are omitted for clarity. The PIO analysis is performed by cutting the Si5-P6 bond. Each PIO pair leads to a bonding PIMO. The PBI quantifies the strength of the interaction. The isosurfaces with the isovalue of 0.050 au are plotted.

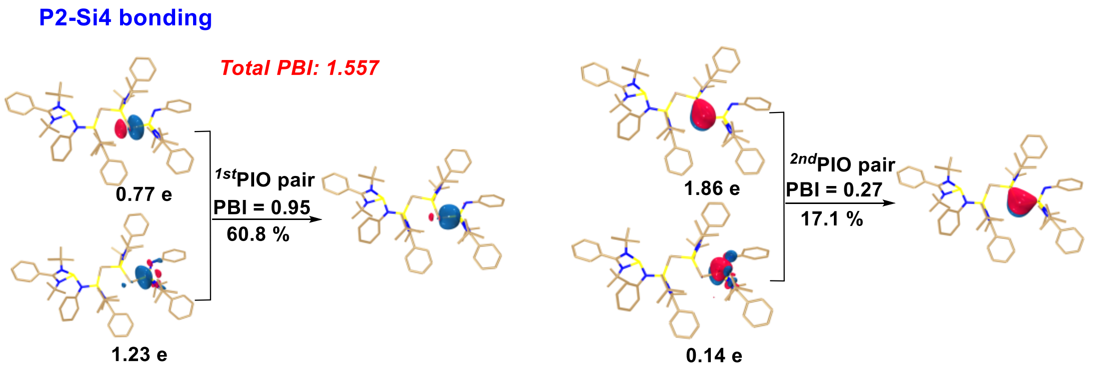

Figure S43. PIO analysis on the bonding modes of P2-Si4 bond in compound **4**. Hydrogen atoms in 3D structures are omitted for clarity. The PIO analysis is performed by cutting the P6-Si7 bond. Each PIO pair leads to a bonding PIMO. The PBI quantifies the strength of the interaction. The isosurfaces with the isovalue of 0.050 au are plotted.

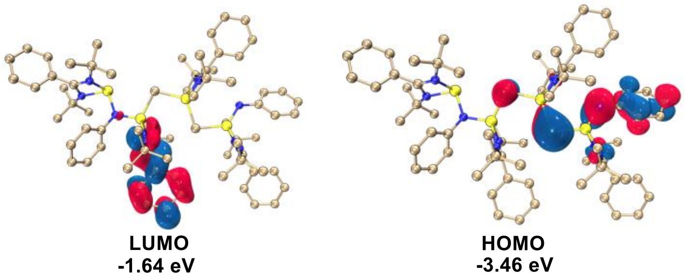

Figure S44. HOMO and LUMO orbitals of the compound **4**. Hydrogen atoms in 3D structures are omitted for clarity. The isosurface 0.050 au is plotted.

Cartesian Coordinates

Compound 2

TPSS-D3BJ/Def2-SVP~ma-TZVP
E = -2937.629024 a.u.

Si	1.37194300	1.15187600	0.26464600
Si	-1.37182500	1.15194800	-0.26531000
P	0.80045600	3.11144000	0.81150800
P	-0.80089200	3.11131600	-0.81327900
N	2.61852500	0.18260900	1.19851600
N	0.00004100	0.09424900	-0.00030600
N	2.75332500	0.68312600	-0.89930500
N	-2.61845600	0.18191000	-1.19852300
N	-2.75309200	0.68373900	0.89900000
C	-0.00003300	-1.31407500	-0.00024100
C	4.80257800	-0.44852900	0.07067300
C	3.42894500	0.11599000	0.12003000
C	-0.91027100	-2.03947200	0.80383500
H	-1.60685800	-1.48193100	1.43588300
C	3.23297400	1.26789000	-2.17555700
C	2.72634800	-0.41671100	2.54725800
C	0.91012800	-2.03971400	-0.80419100
H	1.60685100	-1.48236700	-1.43625700
C	-0.00022300	-4.15249400	-0.00003300
H	-0.00032200	-5.24710800	0.00002600
C	-3.42879300	0.11602100	-0.11997600
C	-0.90930000	-3.43986100	0.79822600

H -1.61954900 -3.98084400 1.43422700
C 1.40067600 -0.08514200 3.25458700
H 1.22317400 1.00389500 3.26103100
H 1.42476200 -0.45401200 4.29391700
H 0.55964200 -0.56601400 2.72919700
C 3.90943000 2.62283700 -1.87815600
H 3.20973800 3.27702400 -1.32989900
H 4.20862300 3.11606400 -2.81963600
H 4.81451800 2.47727600 -1.26282000
C -4.80237300 -0.44861000 -0.07001900
C 1.97140100 1.48360900 -3.02910700
H 1.52141600 0.51409800 -3.30097000
H 2.23000200 2.02149200 -3.95691900
H 1.21447500 2.07362500 -2.47697800
C 0.90896700 -3.44009800 -0.79838100
H 1.61917100 -3.98126000 -1.43428000
C 5.91215700 0.33905100 0.43103800
H 5.75806500 1.36741600 0.77145100
C 3.90148000 0.21840700 3.31763800
H 4.86821500 -0.03343300 2.85182400
H 3.91488300 -0.15657400 4.35575900
H 3.79274400 1.31608900 3.34222300
C 4.19989200 0.32367800 -2.91358300
H 5.16848700 0.22677000 -2.39871300
H 4.39094100 0.72957800 -3.92187600
H 3.75866100 -0.68215700 -3.02570800
C 4.99676000 -1.77303900 -0.36593300
H 4.12966800 -2.37970100 -0.64445600
C 2.89223600 -1.94677600 2.45250600
H 2.07873300 -2.38579600 1.85120100
H 2.85776800 -2.38503800 3.46511900
H 3.85855800 -2.21974000 1.99795700
C -2.72685400 -0.41733300 -2.54723600
C -3.23254400 1.26887600 2.17514800
C 7.20522100 -0.19415900 0.34658800
H 8.06554900 0.42396600 0.62295100
C 6.29084100 -2.30210200 -0.44206400
H 6.43676900 -3.33280500 -0.78122000
C -5.91225400 0.33883500 -0.42973800
H -5.75850100 1.36723300 -0.77020100
C 7.39649900 -1.51347500 -0.08942300
H 8.40794000 -1.92768100 -0.15422500
C -1.40122700 -0.08628200 -3.25491100
H -1.22314500 1.00266700 -3.26109100
H -1.42589700 -0.45482400 -4.29434100
H -0.56028300 -0.56780300 -2.72996600
C -4.19957300 0.32502700 2.91351000
H -5.16820300 0.22804800 2.39871400
H -4.39053500 0.73132400 3.92165800
H -3.75844500 -0.68081300 3.02597600
C -4.99615400 -1.77314700 0.36669200
H -4.12883500 -2.37970500 0.64472700
C -6.29012900 -2.30235600 0.44358300
H -6.43573900 -3.33306400 0.78285800
C -1.97092000 1.48457300 3.02862200
H -1.52122900 0.51504100 3.30091300
H -2.22938000 2.02292600 3.95620300
H -1.21383100 2.07416500 2.47626200
C -3.90879400 2.62386600 1.87746600
H -3.20908300 3.27778100 1.32891700
H -4.20779900 3.11741700 2.81884000
H -4.81397500 2.47829600 1.26226800

C	-7.20520400	-0.19453700	-0.34457300
H	-8.06576000	0.42348200	-0.62046600
C	-7.39608300	-1.51387000	0.09156100
H	-8.40743700	-1.92819800	0.15693600
C	-2.89328900	-1.94734600	-2.45253000
H	-2.07984400	-2.38666500	-1.85135200
H	-2.85912100	-2.38556300	-3.46517200
H	-3.85963800	-2.21999800	-1.99785900
C	-3.90190600	0.21829900	-3.31729500
H	-4.86858900	-0.03270500	-2.85092600
H	-3.91608100	-0.15706700	-4.35527100
H	-3.79243700	1.31590000	-3.34233500

Compound 3
TPSS-D3BJ/Def2-SVP~ma-TZVP
E = -6558.177786 a.u.

P	1.43870100	-0.92291900	1.58229800
Si	1.86021400	1.25715100	0.81619100
P	3.18449400	-2.19853800	1.19800400
P	0.16226700	-1.09046900	-0.24905300
Si	3.86096600	-0.73099700	-0.18110900
P	-0.16261500	1.08998200	-0.24890100
P	-1.43890000	0.92232100	1.58256900
P	-3.18467300	2.19801600	1.19848500
Si	-3.86108100	0.73084500	-0.18105900
Si	-1.86054000	-1.25757200	0.81609700
N	3.38801000	0.94054800	-0.02927300
N	2.00031300	2.26527800	2.33150500
N	1.77373000	3.29253900	0.43073700
N	5.65895100	-0.79073600	-0.55601200
N	4.08999000	-1.12653000	-2.00144700
N	-3.38829900	-0.94077500	-0.02935500
N	-1.77412500	-3.29311700	0.43019800
N	-4.08983300	1.12674100	-2.00135400
N	-2.00034400	-2.26600000	2.33111300
N	-5.65902100	0.79095000	-0.55615300
C	4.31393900	1.95094900	-0.42212900
C	4.81877400	2.02369300	-1.73718300
H	4.45067200	1.31547100	-2.48206700
C	1.67558600	3.45380000	1.74005400
C	5.41654300	-1.21390500	-1.81612900
C	4.78277000	2.88790900	0.52361400
H	4.39805000	2.83404500	1.54466800
C	-1.67570000	-3.45448400	1.73945700
C	-4.31430100	-1.95106800	-0.42232200
C	6.88267200	-0.84462600	0.27785600
C	1.08880700	4.60516500	2.48023200
C	5.76828400	2.99219300	-2.09018600
H	6.14042600	3.03004800	-3.12057100
C	-0.22304700	4.44414400	2.97178000
H	-0.75251700	3.50244800	2.78748000
C	-5.41637100	1.21446900	-1.81611300
C	1.35989900	4.22357800	-0.65042500
C	2.37319200	1.96926700	3.74134800
C	6.23070900	3.91649600	-1.14180800
H	6.97036700	4.67449000	-1.41897500
C	5.72627500	3.85832800	0.16719800
H	6.07380300	4.57290400	0.92172800
C	1.77325300	5.81400100	2.69768600
H	2.80129200	5.92788900	2.34131500
C	3.53269400	0.95317200	3.69104100

H	3.27007300	0.05611900	3.10410100
H	3.79040100	0.62759500	4.71359100
H	4.42766500	1.40693700	3.23432100
C	-6.42313100	1.72349600	-2.78284300
C	-1.08853400	-4.60578900	2.47942800
C	-0.10163900	4.69294000	-0.48515400
H	-0.77474100	3.83329100	-0.32144500
H	-0.42695600	5.22050400	-1.39915800
H	-0.21432800	5.38421300	0.36472100
C	-4.81942900	-2.02341000	-1.73728400
H	-4.45146500	-1.31499600	-2.48205300
C	6.42352300	-1.72232900	-2.78295200
C	-4.78292500	-2.88832400	0.52323000
H	-4.39800300	-2.83476700	1.54422000
C	-0.84547100	5.49332200	3.65858000
H	-1.87036700	5.36499200	4.02146500
C	2.31751200	5.43483000	-0.67731600
H	2.20208800	6.05702200	0.22363700
H	2.09064000	6.06554700	-1.55509200
H	3.36390100	5.09456300	-0.74820000
C	3.20307400	-1.41309400	-3.15249900
C	-2.37357300	-1.97037000	3.74095600
C	-1.36030300	-4.22399300	-0.65110000
C	1.51522600	3.47264400	-1.98833900
H	2.54843600	3.11149700	-2.10683200
H	1.28438000	4.15981400	-2.82058700
H	0.82855500	2.61302600	-2.04361700
C	-1.51562000	-3.47284200	-1.98889900
H	-2.54877800	-3.11148700	-2.10723200
H	-1.28498300	-4.15993300	-2.82127100
H	-0.82879600	-2.61334000	-2.04411500
C	1.14972900	6.85532800	3.39789400
H	1.69033600	7.79150000	3.57271100
C	6.50164500	-0.19282200	1.61974000
H	6.21790300	0.86109100	1.46452500
H	7.36051600	-0.22974700	2.31101800
H	5.64945300	-0.72483800	2.07691500
C	2.87562100	3.22347200	4.48231700
H	3.64826500	3.75209900	3.89583100
H	3.33121100	2.90261600	5.43501600
H	2.06606200	3.93083800	4.71559700
C	-7.19432600	0.82603800	-3.54375900
H	-7.05926200	-0.25096500	-3.40520500
C	-6.58736700	3.11096100	-2.95436300
H	-5.98060100	3.80338700	-2.36241800
C	-5.76904500	-2.99177700	-2.09036600
H	-6.14141900	-3.02930600	-3.12067900
C	2.14546600	-0.29100400	-3.18457000
H	1.63414900	-0.20290000	-2.21231000
H	1.37975800	-0.51976400	-3.94500700
H	2.60515600	0.68048000	-3.42956100
C	0.22329600	-4.44436600	2.97092000
H	0.75236100	-3.50239700	2.78683700
C	7.29676700	-2.31155500	0.51166200
H	6.45920900	-2.87178700	0.96210000
H	8.16038000	-2.35669800	1.19808300
H	7.58579100	-2.79895900	-0.43454600
C	8.02171300	-0.03589800	-0.37239700
H	8.39922100	-0.52134000	-1.28630500
H	8.86199000	0.04767000	0.33854900
H	7.67086100	0.97976500	-0.62308300
C	-0.16052000	6.69914600	3.87493400

H	-0.64696000	7.51739500	4.41644500
C	-3.20284300	1.41301700	-3.15242100
C	-6.88256700	0.84585000	0.27792900
C	-7.52055900	3.59468000	-3.88035500
H	-7.64832900	4.67443000	-4.00861000
C	7.19423800	-0.82442700	-3.54382700
H	7.05863200	0.25249700	-3.40519300
C	-2.31797500	-5.43519600	-0.67814700
H	-2.20261000	-6.05746800	0.22276000
H	-2.09110800	-6.06585000	-1.55596900
H	-3.36434200	-5.09486700	-0.74902500
C	0.10121400	-4.69344700	-0.48592700
H	0.77434100	-3.83384800	-0.32206300
H	0.42649700	-5.22083300	-1.40004500
H	0.21389800	-5.38489200	0.36380600
C	2.52493700	-2.77469000	-2.89764600
H	3.27925700	-3.57957300	-2.85825400
H	1.80546500	-3.00062700	-3.70410300
H	1.98536100	-2.75164000	-1.93786500
C	1.17178600	1.36301600	4.48234000
H	0.32544400	2.06833200	4.50106500
H	1.44793200	1.10708500	5.52074300
H	0.84978300	0.44882700	3.96289100
C	6.58841600	-3.10970100	-2.95462800
H	5.98203000	-3.80247900	-2.36270800
C	-5.72651900	-3.85862100	0.16673100
H	-6.07387100	-4.57342900	0.92112200
C	3.94769900	-1.42362300	-4.49913900
H	4.51470200	-0.48928500	-4.65123300
H	3.19789700	-1.50317000	-5.30466600
H	4.64096400	-2.27298600	-4.59557100
C	8.11922600	-1.31363800	-4.47458600
H	8.71293600	-0.61230800	-5.06984000
C	-3.94751000	1.42385600	-4.49903900
H	-4.51493000	0.48976800	-4.65110400
H	-3.19769800	1.50305600	-5.30459000
H	-4.64040400	2.27352500	-4.59545600
C	-6.23126500	-3.91637200	-1.14217400
H	-6.97100300	-4.67426400	-1.41940900
C	-6.50224100	0.19238600	1.61920600
H	-6.21994600	-0.86178600	1.46309800
H	-7.36094200	0.22994900	2.31066300
H	-5.64927800	0.72291400	2.07667800
C	-8.28467700	2.69881300	-4.64317200
H	-9.00988600	3.07882200	-5.37013800
C	-8.11912500	1.31578500	-4.47441700
H	-8.71321000	0.61480100	-5.06970600
C	-3.53315300	-0.95435800	3.69067200
H	-3.27041000	-0.05706000	3.10415900
H	-3.79120900	-0.62920000	4.71326800
H	-4.42794100	-1.40800800	3.23348200
C	-1.77245300	-5.81497000	2.69662100
H	-2.80048600	-5.92919200	2.34034600
C	-1.17240100	-1.36417400	4.48237600
H	-0.32597900	-2.06939300	4.50112100
H	-1.44880600	-1.10853500	5.52078400
H	-0.85039100	-0.44981300	3.96323500
C	8.28542600	-2.69656600	-4.64349800
H	9.01077300	-3.07615400	-5.37054500
C	-2.14562400	0.29057000	-3.18456700
H	-1.63445900	0.20211200	-2.21226100
H	-1.37974100	0.51920800	-3.94486500

H	-2.60560700	-0.68071600	-3.42979700
C	0.84623300	-5.49348000	3.65734800
H	1.87111600	-5.36481900	4.02015400
C	-7.29447300	2.31320500	0.51297600
H	-6.45602300	2.87183400	0.96374600
H	-8.15794800	2.35910200	1.19952300
H	-7.58286500	2.80175200	-0.43284200
C	-8.02291700	0.03935000	-0.37277600
H	-8.40000000	0.52612300	-1.28614500
H	-8.86310400	-0.04365200	0.33833900
H	-7.67360700	-0.97660300	-0.62445000
C	7.52177600	-3.59287800	-3.88073000
H	7.65004600	-4.67255300	-4.00911200
C	-2.52421500	2.77437300	-2.89756800
H	-3.27826700	3.57948300	-2.85788500
H	-1.80488600	3.00017100	-3.70419800
H	-1.98437900	2.75103400	-1.93793800
C	0.16181100	-6.69965400	3.87341800
H	0.64864800	-7.51786700	4.41462600
C	-2.87604200	-3.22482800	4.48146300
H	-3.64849700	-3.75337100	3.89464600
H	-3.33187700	-2.90430300	5.43415500
H	-2.06645700	-3.93217500	4.71470700
C	-1.14842800	-6.85623300	3.39647700
H	-1.68863800	-7.79267200	3.57109400

Cartesian Coordinates
Compound 4
TPSS-D3BJ/Def2-SVP~ma-TZVP
E = -5192.458127 a.u.

P	-1.76750500	-0.46810600	-0.14755700
Si	2.03109100	-0.58065500	0.17198000
Si	-0.71889700	1.39419800	0.02839000
P	1.46398600	1.46672400	0.17424100
Si	-3.84314400	0.14462900	-0.11074100
Si	4.61215500	0.78821700	0.88195300
N	1.27288600	-1.91848300	1.20414300
N	-4.89113500	-1.00362600	0.95591400
N	1.46321300	-1.92669200	-0.94501600
N	5.80882200	1.29407000	-0.51948700
N	3.76755100	-0.66638900	0.21426200
N	6.33469900	0.05321300	1.15742300
N	-1.23412900	2.80236600	-1.06466400
N	-4.79688700	-1.07618200	-1.20237400
N	-1.35959500	2.76984300	1.08538000
N	-4.40739800	1.68676400	-0.02729000
C	0.97679000	-2.62618100	0.09822000
C	0.28863600	-3.94427000	0.04530000
C	-5.38432500	-1.62511000	-0.12420600
C	4.46719400	-1.80764800	-0.25673300
C	1.00588500	-2.15007700	2.64309000
C	6.85801000	0.79429300	0.16309600
C	8.29991300	1.08866200	-0.07307600
C	5.36497300	-1.71542700	-1.34459000
H	5.50128500	-0.74416300	-1.82380800
C	-1.61459700	3.52426100	0.00161300
C	-5.74332400	2.01618500	0.06201900
C	1.43797900	-2.17518900	-2.40624000
C	1.05362700	-5.12409200	-0.03510600
H	2.14510500	-5.05952700	-0.06314400
C	-5.34052600	-1.01044800	2.36435500

C	-1.11580600	-4.01630400	0.08896200
H	-1.68976300	-3.08434100	0.14124200
C	-2.24209500	4.87136100	-0.01286900
C	2.09331000	-0.93318500	-3.03402800
H	1.55093900	-0.02032300	-2.74048300
H	2.09081100	-1.01800600	-4.13341500
H	3.13640500	-0.84034000	-2.69053800
C	-6.43077300	-2.68278600	-0.14132100
C	5.72698500	2.33216100	-1.56749000
C	4.29414400	-3.06918900	0.35510100
H	3.60649600	-3.14798700	1.19999200
C	-1.30516400	3.12115300	2.51783900
C	6.98398900	-0.71493300	2.23628900
C	-4.67545800	-1.58191300	-2.58479000
C	-1.74655600	-5.26633900	0.04376600
H	-2.83982100	-5.31899500	0.06696300
C	0.41329900	-6.36919300	-0.07201500
H	1.01142200	-7.28432000	-0.13186900
C	-0.98746600	-6.44288200	-0.03478400
H	-1.48579200	-7.41739100	-0.06806000
C	6.06428700	-2.84264400	-1.79426300
H	6.75220700	-2.74501100	-2.64195500
C	1.81266900	-1.06371300	3.37867700
H	2.89051300	-1.18199800	3.18120900
H	1.64144400	-1.13304600	4.46569900
H	1.51511600	-0.06010900	3.03204800
C	2.26421400	-3.42709800	-2.76390400
H	3.28199700	-3.35907600	-2.34779600
H	2.33844300	-3.51125900	-3.86231800
H	1.78334000	-4.34403700	-2.38746900
C	-0.49655100	-1.99934300	2.94937200
H	-0.88578000	-1.06403800	2.51995400
H	-0.65662500	-2.00424400	4.04180000
H	-1.07264600	-2.83092800	2.51140200
C	8.89655900	2.24549400	0.46400300
H	8.30097500	2.91934000	1.08774400
C	-1.38945800	3.04714600	-2.51264700
C	-3.76997500	-2.83149700	-2.58667400
H	-2.80427200	-2.58951200	-2.11495300
H	-3.58460100	-3.17788800	-3.61886200
H	-4.24365100	-3.65694200	-2.02812000
C	-3.64679000	4.94262900	-0.05756800
H	-4.21191700	4.00676600	-0.08786100
C	-6.21262600	2.87881300	1.10011800
H	-5.48396600	3.25806400	1.82377100
C	-0.01018400	-2.32096100	-2.90505300
H	-0.46936900	-3.24629500	-2.52011400
H	-0.02099300	-2.36765300	-4.00793400
H	-0.61930300	-1.46723700	-2.56532600
C	-6.10280800	-4.03437200	0.06920700
H	-5.06362900	-4.31232700	0.26884200
C	4.98877700	-4.19638700	-0.10305900
H	4.83789800	-5.16177100	0.39373200
C	9.06878300	0.21546300	-0.86519100
H	8.59850300	-0.67848100	-1.28700900
C	-1.03035800	1.80095100	3.25597400
H	-1.84461200	1.08410600	3.07444900
H	-0.94251000	1.98008300	4.34075000
H	-0.08812400	1.35936600	2.89015200
C	-7.76957600	-2.31829900	-0.38651200
H	-8.01587700	-1.26366600	-0.54520300
C	5.84044500	-1.22804300	3.13062100

H	5.26123300	-0.38150200	3.53908900
H	6.24618500	-1.81732400	3.97014700
H	5.16054800	-1.86921600	2.54704700
C	5.88156800	-4.09246000	-1.18078900
H	6.42700000	-4.97193500	-1.53741100
C	1.49498700	-3.54292300	3.08815800
H	0.93686500	-4.35043200	2.58820300
H	1.34401800	-3.64726900	4.17637900
H	2.57115700	-3.67334500	2.88081600
C	-5.11643000	-2.40404000	2.98689400
H	-5.77681100	-3.16032200	2.53219000
H	-5.33372800	-2.36934100	4.06891900
H	-4.06686000	-2.71822200	2.85064100
C	-4.44775800	0.01725300	3.07958500
H	-3.38946300	-0.28057400	2.99431500
H	-4.71611800	0.08154400	4.14740800
H	-4.56537600	1.00906000	2.61422200
C	-2.64195700	3.71819700	2.99478200
H	-2.81803100	4.71878600	2.56938900
H	-2.63374400	3.80855800	4.09512700
H	-3.47198300	3.05961900	2.69404800
C	-0.73474900	1.83935100	-3.20659100
H	0.33292300	1.78203900	-2.93564600
H	-0.82403700	1.93498700	-4.30176500
H	-1.21704200	0.90204800	-2.88434300
C	4.39001500	2.09117300	-2.29542200
H	4.41166300	1.13067000	-2.83673000
H	4.20219700	2.89711300	-3.02578500
H	3.54588800	2.06174000	-1.58265000
C	-1.47308100	6.04889800	0.02414000
H	-0.38053000	5.98742300	0.04604900
C	-6.04447700	-1.90651900	-3.21498900
H	-6.51532300	-2.78835400	-2.75330900
H	-5.90547800	-2.11891900	-4.28952600
H	-6.73202600	-1.04914900	-3.11772200
C	-3.51479800	7.36888300	-0.01129500
H	-4.01048800	8.34562600	-0.00745000
C	-7.10305900	-5.01508000	0.02597400
H	-6.84106900	-6.06607600	0.18655700
C	6.88038600	2.22868700	-2.58432500
H	7.84922000	2.52699100	-2.15477700
H	6.66588000	2.89646900	-3.43660100
H	6.97137100	1.19823000	-2.96975100
C	-8.43499600	-4.65045400	-0.21855700
H	-9.21601700	-5.41727200	-0.24985500
C	-2.88286300	3.14309700	-2.88725100
H	-3.44990800	2.33440800	-2.39906100
H	-3.00236600	3.07633500	-3.98305300
H	-3.31261900	4.10125300	-2.55304600
C	-4.27830900	6.19218800	-0.05491100
H	-5.37187800	6.23852100	-0.08273700
C	-4.00168100	-0.45195100	-3.38245200
H	-4.64213400	0.44523600	-3.40527100
H	-3.80823700	-0.77736700	-4.41859400
H	-3.04303000	-0.18136400	-2.90878900
C	-6.81910100	-0.58675700	2.48173700
H	-6.98553200	0.37830000	1.97530300
H	-7.08863300	-0.47677700	3.54694700
H	-7.48797800	-1.34329200	2.04042400
C	-7.56382500	3.22358300	1.22202700
H	-7.87580100	3.87934100	2.04426700
C	-6.73447500	1.55843900	-0.86102000

H	-6.40924000	0.91844000	-1.68692300
C	-8.76567300	-3.30142600	-0.42037100
H	-9.80512000	-3.01251100	-0.60705100
C	10.41713200	0.49890000	-1.11971500
H	11.00838000	-0.18321000	-1.73951600
C	10.24489000	2.52541900	0.20777500
H	10.70263600	3.42639700	0.62944900
C	-0.13595100	4.09714500	2.76870500
H	0.80600400	3.66014600	2.39434900
H	-0.03224900	4.29536600	3.85031000
H	-0.31201100	5.05983900	2.26101600
C	11.00676500	1.65402200	-0.58562500
H	12.06014600	1.87539500	-0.78662400
C	-2.11372600	7.29575200	0.02587300
H	-1.51555500	8.21256700	0.05590000
C	-0.63530900	4.32904300	-2.92167300
H	-1.08918900	5.22489100	-2.46846700
H	-0.67227300	4.44564300	-4.01923500
H	0.42167200	4.26704600	-2.60928400
C	-8.51988900	2.73567200	0.31595700
H	-9.57652300	3.00494900	0.41568400
C	5.71270700	3.72890000	-0.90873100
H	4.87971800	3.79651800	-0.18846800
H	5.58730000	4.51582100	-1.67359900
H	6.65855400	3.91617900	-0.37220000
C	7.93002200	0.16699300	3.07838300
H	8.79934400	0.50380500	2.49137500
H	8.30599000	-0.40760600	3.94333400
H	7.39189300	1.05389900	3.45555500
C	-8.08395300	1.90446600	-0.73040200
H	-8.80765900	1.52550600	-1.46373400
C	7.74837900	-1.91506200	1.64029000
H	7.08051800	-2.51887700	1.00475000
H	8.13924800	-2.55345400	2.45230100
H	8.60352100	-1.57533900	1.03352100

C References

1. Fink, W. Beitraege zur Chemie der Si-N-Bindung, VII. N,N’-alkylierte und -arylierte cyclodisilazane. *Helvetica Chimica Acta*. **1964**, *47*, Fasciculus 2, 58.

2. Sen, S. S.; Roesky, H. W.; Stern, D.; Henn, J.; and Stalke, D. High Yield Access to Silylene RSiCl (R = PhC(N*t*Bu)$_2$) and Its Reactivity toward Alkyne: Synthesis of Stable Disilacyclobutene. *J. Am. Chem. Soc*. **2010**, *132*, 1123-1126.

3. Sheldrick, G. M. *SHELX-97 Program for Crystal Structure Determination*, Universität Göttingen, Germany (1997).

4. Frisch, M. J.; Trucks, G. W.; Schlegel, H. B.; Scuseria, G. E.; Robb, M. A.; Cheeseman, J. R.; Scalmani, G.; Barone, V.; Mennucci, B.; Petersson, G. A.; Nakatsuji, H.; Caricato, M.; Li, X.; Hratchian, H. P.; Izmaylov, A. F.; Bloino, J.; Zheng, G.; Sonnenberg, J. L.; Hada, M.; Ehara, M.; Toyota, K.; Fukuda, R.; Hasegawa, J.; Ishida, M.; Nakajima, T.; Honda, Y.; Kitao, O.; Nakai, H.; Vreven, T.; Montgomery, J. A., Jr.; Peralta, J. E.; Ogliaro, F.; Bearpark, M.; Heyd, J. J.; Brothers, E.; Kudin, K. N.; Staroverov, V. N.; Kobayashi, R.; Normand, J.; Raghavachari, K.; Rendell, A.; Burant, J. C.; Iyengar, S. S.; Tomasi, J.; Cossi, M.; Rega, N.; Millam, J. M.; Klene, M.; Knox, J. E.; Cross, J. B.; Bakken, V.; Adamo, C.; Jaramillo, J.; Gomperts, R.; Stratmann, R. E.; Yazyev, O.; Austin, A. J.; Cammi, R.; Pomelli, C.; Ochterski, J. W.; Martin, R. L.; Morokuma, K.; Zakrzewski, V. G.; Voth, G. A.; Salvador, P.; Dannenberg, J. J.; Dapprich, S.; Daniels, A. D.; Farkas, O.; Foresman, J. B.; Ortiz, J. V.; Cioslowski, J.; Fox, D. J., Gaussian 16, Revision A.03; Gaussian, Inc., Wallingford CT, **2016**.

5. Tao, J. M.; Perdew, J. P.; Staroverov, V. N.; Scuseria. G. E. Climbing the density functional ladder: Nonempirical meta-generalized gradient approximation designed for molecules and solids. *Phys. Rev. Lett.* **2003**, *91*, 146401.

6. Weigend F.; Ahlrichs. R. Balanced basis sets of split valence, triple zeta valence and quadruple zeta valence quality for H to Rn: Design and assessment of accuracy. *Phys. Chem. Chem. Phys.* **2005**, *7*, 3297–305.

7. Zheng, J.; Xu, X.; Truhlar, D. G. Minimally augmented Karlsruhe basis sets. *Theor. Chem. Acc.* **2010**, *128*, 295-305.

8. Papajak, E.; Zheng, J.; Xu, X.; Leverentz, H. R.; Truhlar, D. G. Perspectives on Basis Sets Beautiful: Seasonal Plantings of Diffuse Basis Functions. *J. Chem. Theory. Comput.* **2011**, *7*, 3027-3034.

9. Wilson, P. J.; Bradley, T. J.; Tozer, D. J. Hybrid exchange-correlation functional determined from thermochemical data and ab initio potentials. *J. Chem. Phys.* **2001**, *115*, 9233-9242

10. Weigend, F.; Ahlrichs, R. Balanced basis sets of split valence, triple zeta valence and quadruple zeta valence quality for H to Rn: Design and assessment of accuracy. *Phys. Chem. Chem. Phys.* **2005**, *7*, 3297-3305.

11. Jameson, C. J.; De Dios, A.; Keith Jameson, A. Absolute shielding scale for ^{31}P from gas-phase NMR studies. *Chem. Phys. Lett.* **1990**, *167*, 575-582.

12. Legault, C. Y.; CYL view, 1.0b ed.; Université de Sherbrooke: Sherbrooke, Québec, Canada, **2009**; http://www.cylview.org.

13. Humphrey, W.; Dalke, A.; Schulten, K. VMD: Visual molecular dynamics. *J. Mol. Graphics.* **1996**, *14*, 33-38.

14. Lu, T.; Chen, F.; Multiwfn: a multifunctional wavefunction analyzer. *J. Comput. Chem*. **2012**, *33*, 580-592.

15. Fallah-Bagher-Shaidaei, H.; Wannere, C. S.; Corminboeuf, C.; Puchta R.; Schleyer, P. v. R. Which NICS aromaticity index for planar p rings is best? *Org. Lett*. **2006**, *8*, 863-866.

16. Simeth, N. A.; Bellisario, A.; Crespi, S.; Fagnoni, M.; König, B. Substituent Effects on 3-Arylazoindole Photoswitches. *J. Org. Chem.* **2019**, *84*, 6565−6575.
